# Candidate disease gene prediction using *Gentrepid*: application to a genome-wide association study on coronary artery disease

**DOI:** 10.1002/mgg3.40

**Published:** 2013-11-13

**Authors:** Sara Ballouz, Jason Y Liu, Martin Oti, Bruno Gaeta, Diane Fatkin, Melanie Bahlo, Merridee A Wouters

**Affiliations:** 1 Structural and Computational Biology Division, Victor Chang Cardiac Research InstituteDarlinghurst, NSW, 2010, Australia; 2 School of Computer Science and Engineering, University of New South WalesKensington, NSW, 2052, Australia; 3 Centre for Molecular and Biomolecular Informatics, Radboud University Nijmegen Medical CentreNijmegen, The Netherlands; 4 School of Medical Sciences, University of New South WalesKensington, NSW, 2052, Australia; 5 Molecular Cardiology and Biophysics Division, Victor Chang Cardiac Research InstituteDarlinghurst, NSW, 2010, Australia; 6 Bioinformatics Division, The Walter and Eliza Hall Institute of Medical ResearchParkville, VIC, 3052, Australia; 7 School of Medicine, Deakin UniversityGeelong, VIC, 3217, Australia; 8 School of Life and Environmental Sciences, Deakin UniversityGeelong, VIC, 3217, Australia

**Keywords:** Candidate gene prediction, cis-ruption, complex diseases, coronary artery disease, genome-wide association study, miRNA, Wellcome Trust Case Control Consortium, WTCCC

## Abstract

Current single-locus-based analyses and candidate disease gene prediction methodologies used in genome-wide association studies (GWAS) do not capitalize on the wealth of the underlying genetic data, nor functional data available from molecular biology. Here, we analyzed GWAS data from the Wellcome Trust Case Control Consortium (WTCCC) on coronary artery disease (CAD). *Gentrepid* uses a multiple-locus-based approach, drawing on protein pathway- or domain-based data to make predictions. Known disease genes may be used as additional information (*seeded* method) or predictions can be based entirely on GWAS single nucleotide polymorphisms (SNPs) (*ab initio* method). We looked in detail at specific predictions made by *Gentrepid* for CAD and compared these with known genetic data and the scientific literature. *Gentrepid* was able to extract known disease genes from the candidate search space and predict plausible novel disease genes from both known and novel WTCCC-implicated loci. The disease gene candidates are consistent with known biological information. The results demonstrate that this computational approach is feasible and a valuable discovery tool for geneticists.

## Introduction

Coronary artery disease (CAD) is the leading cause of death and disability in the world (Lopez et al. 2006). Also known as coronary heart disease, it involves narrowing of the arteries and small blood vessels that supply blood and oxygen to the heart, and is typically caused by the build-up of plaque. Multiple risk factors have been identified for CAD that include family history, lipid levels, hypertension, smoking, and diabetes (Swerdlow et al. 2012). Heritability of CAD has been calculated to be between 40% and 50%, but only ∼10% is explained by genetic variations discovered to date (Peden and Farrall 2011). The likely reason for the missing heritability is the complex nature of the disease. Multiple genes and environmental factors contribute to the phenotype, and causative alleles may have small effects that are not detected by the current methods used, such as genome-wide association studies (GWAS). GWAS are designed to detect genetic risk factors of complex diseases and quantitative traits that are reasonably common in a population through the assessment of correlations between genetic variants such as single nucleotide polymorphisms (SNPs) and trait differences. Unfortunately, rarer variants that are not directly represented on the genotyping array, as well as common variants with more modest effects, are harder to detect because a highly stringent significance threshold is used to correct for the number of false positives (Pearson and Manolio 2008). SNPs that do not achieve genome-wide statistical significance in these studies may still be of importance.

Following detection of an association signal between a SNP and the phenotype, the next step of identifying the causal genetic basis is nontrivial for two reasons. First, the SNP is most likely in linkage disequilibrium (LD) with the true variant, as SNP chips contain only a selection of common variants and have incomplete coverage of the genome. Second, even with knowledge of the true variant, its functional significance may not be obvious as the genetic architecture of the genome still remains unclear. GWAS typically report the nearest neighboring gene to the disease-associated SNP/locus, but this assumption may not hold for all reported associations. For instance, long range regulation and distal control elements suggest the disease gene may be near the significant SNP but may not be the closest gene to it (Kikuta et al. 2007). Further to this, in work on simulated GWAS data, it was found that synthetic associations can be created by rarer alleles up to 2 Mbp from the true association signal (Dickson et al. 2010), essentially lowering the resolution of the association locus discovery. In recent work derived from ENCODE, most variants from GWAS were shown to be concentrated in regulatory regions of the DNA (Maurano et al. 2012), with 40% enrichment of SNPs in Deoxyribonuclease I (DNase I) hypersensitive sites (DHS), and up to 76.6% in LD with a DHS. Around 40% of genes linked with a DHS are over 250 Kbp away and not in LD with the SNP in the DHS.

In summary, the two main challenges in analyzing GWAS data are the high false negative rate for genotype–phenotype association, and low disease gene discovery rate. With these shortcomings of GWAS in mind, we previously proposed a bioinformatic strategy to sift through candidate genes near a larger number of SNPs by lowering the significance threshold (Ballouz et al. 2011). The increased number of genetic loci can be dealt with by automated candidate gene prediction and prioritization systems. There are currently many bioinformatic tools available to predict and prioritize gene candidates which have been reviewed elsewhere (Oti et al. 2011; Moreau and Tranchevent 2012), each with varying underlying data sources, inputs, algorithms, and ranking strategies. Several tools have been adapted to allow the prioritization of candidates from GWAS data (Holmans et al. 2009; Raychaudhuri et al. 2009; Duncan et al. 2010; Wang et al. 2010). In this study, we used the candidate disease gene prediction tool, *Gentrepid*, which uses two general approaches: a systems biology approach, looking at pathway data and protein–protein interaction (PPI) data; along with a novel functional approach whereby protein domains parsed in sequences are used to infer function. Although previous pathway analyses of the Wellcome Trust Case Control Consortium (WTCCC) (2007) study data has shown that including biomolecular information identifies numerous known and novel pathways (Torkamani et al. 2008; Elbers et al. 2009), no domain-based homology analysis has yet been performed on this dataset.

Because *Gentrepid* looks at interactions and similarities between loci, it is particularly apt for analyzing the multiple loci suggested by GWAS data. By looking at the GWAS data holistically and incorporating protein information, interactions and common features between loci can be detected, thereby improving candidate disease gene prediction outcomes. *Gentrepid* was originally benchmarked (George et al. 2006) on a standard set of oligogenic diseases with Mendelian inheritance from Turner et al. (2003). It was later benchmarked against other candidate gene prediction systems using GWAS data on type II diabetes from the WTCCC (Wellcome Trust Case Control Consortium 2007) and DIAGRAM (Zeggini et al. 2007) studies (Teber et al. 2009). More recently, we performed an assessment of the system's ability to predict candidate disease genes from GWAS data using several analysis protocols (Ballouz et al. 2011) and compared the results to the popular tools GRAIL (Raychaudhuri et al. 2009) and WebGestalt (Duncan et al. 2010).

Here, we demonstrate use of *Gentrepid* as a discovery tool to select and prioritize valid disease candidates from the CAD WTCCC GWAS (Wellcome Trust Case Control Consortium 2007). Compared to the Framingham study (de las Fuentes et al. 2012) and other meta-analyses, a number of interesting novel genes are identified, some in previously associated loci, which may be valuable to pursue in further genetic and biochemical analyses.

## Materials and Methods

### Data sourcing

**Figure 1:**
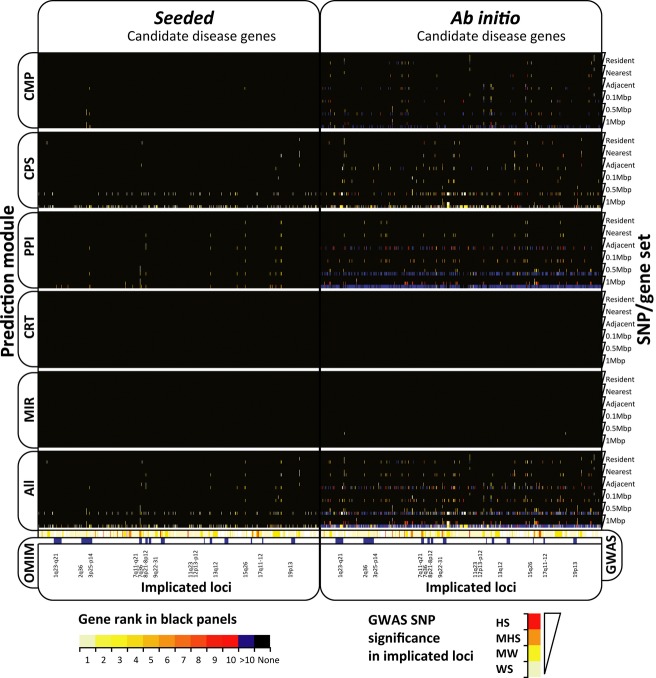
Candidate disease gene prediction and prioritization heatmap for coronary artery disease (CAD) across the combined gene search spaces. Panels on the left are *seeded* predictions made with known disease gene properties. Panels on the right are *ab initio* predictions. Prediction modules used for each panel are annotated on the left, from CPS on the top, followed by CMP, PPI, CRT, and MIR, to the combined predictions shown on the bottom panel. Within each of the 12 panels, the autosomes run along the x-axis, from 1 (left) to 22 (right), and the six gene search spaces investigated run along the y-axis (annotated on the right), each sub divided from HS (top of wedge) to WS (bottom of wedge). The gene ranking key is shown on the bottom left. The lightest colors represent highly prioritized genes, while black signifies no prediction or rank. Below the gene predictions, the original GWAS SNP loci, colored by significance (key on bottom left), are compared to the OMIM loci (blue).

For the genotype data, we obtained SNP association summary statistics from the WTCCC (Wellcome Trust Case Control Consortium 2007) case–control studies of CAD. We mapped these SNPs to 489,763 autosomal SNPs on the genome assembly (build 36.3), of which 459,231 SNPs were retained following WTCCC quality control (Wellcome Trust Case Control Consortium 2007). For genotype–phenotype relationship data, we extracted known CAD disease genes and loci from the Online Mendelian Inheritance in Man (OMIM) database (Hamosh et al. 2002). We queried the Morbid Map flat file by performing a text search for the disease name or parts thereof: “Coronary artery disease”, “coronary heart disease”, and “coronary”. The results were then manually filtered, removing duplicate loci and merging adjacent loci. The final list of known loci consisted of 19 cytogenetic bands and 13 genes (Fig. 1).

### Data preprocessing

We selected four SNP sets by iteratively lowering the stringency threshold of the Cochran-Armitage *P*-value of statistical significance for an association from the original threshold used in the study. These were a highly significant SNP set (HS, *P*_GWA_ <5 × 10^−7^), a moderately high significant set (MHS, *P*_GWA_ ≤10^−5^), a moderately weak significant set (MWS, *P*_GWA_ ≤10^−4^), and a weakly significant set (WS, *P*_GWA_ ≤10^−3^). SNPs in close proximity (within 50 Kbp) were merged into a single locus.

For each of the four significant SNP sets, we created six gene search spaces, three based on SNP gene proximity, labeled the “nearest neighbor” (NN) approach (adjacent, nearest, and resident sets), and three based on SNP gene distance labeled the “bystander” (BY) approach (1 Mbp, 0.5 Mbp, and 0.1 Mbp sets). For the NN approach, the resident set includes only genes with significant SNPs within the gene boundary. The nearest set contains the closest gene to each SNP. In the adjacent set, a gene is selected upstream and downstream of each SNP on both strands of DNA, resulting mostly in four genes for each SNP. For the BY approach, genes were pooled from an interval around each SNP of window sizes of 1 Mbp, 0.5 Mbp, and 0.1 Mbp, respectively. As most SNPs on the chip used by the WTCCC are in noncoding regions, creating several different search spaces ensured that likely genes were included in the analysis. The methods are fully described in the protocol development paper (Ballouz et al. 2011) and a workflow diagram is provided (Fig. S1).

### *Gentrepid* data analysis and validation

We analyzed the data with the *Gentrepid* system, via an in-house database and local standard database queries written in structured query language. We used two modes of input: one that utilizes known disease gene information as seeds (*seeded*); and one that uses only genes within the search space (*ab initio*). For *seeded* mode, we used 13 genes already associated with the disease listed in OMIM (Table 1). We used the original three modules employed by the system to predict and prioritize candidates: two systems biology methods, common pathway scanning (CPS), a pathway-based approach and PPI, a PPI method; and common module profiling (CMP), a domain-based homology approach. The systems biology methods are based on the assumption that common phenotypes are likely to be associated with proteins that partake in the same complex or pathway (Badano and Katsanis 2002; Goh et al. 2007). CMP is a technique based on the principle that candidate genes have similar functions to disease genes already determined for the phenotype (Jimenez-Sanchez et al. 2001). These methods are described in detail in previous work (George et al. 2006; Ballouz et al. 2011).

**Table 1:** Coronary artery disease validation sets.

			Search space set						Significance level			
Gene accession (OMIM)	Genes names (HGNC)	Gene IDs (Entrez)
			1 Mbp	0.5 Mbp	0.1 Mbp	A	N	R	HS	MHS	MWS	WS
OMIM												
601470	*CX3CR1*	1524	X	X		X					X	X
147545	*IRS1*	3667	X			X					X	X
152200	*LPA*	4018	X	X								X
603507	*LRP6*	4040	X									X
163729	*NOS3*	4846	X	X								X
173510	*CD36*	948	X	X		X					X	X
600046	*ABCA1*	19										
600660	*MEF2A*	4205										
158105	*CCL2*	6347										
604824	*KL*	9365										
168820	*PON1*	5444										
602447	*PON2*	5445										
185250	*MMP3*	4314										
WTCCC												
605009	*ADAMTS7*	11173	X	X	X	X	X	X			X	X
600160	*CDKN2A*	1029	X	X	X				X	X	X	X
600431	*CDKN2B*	1030	X	X	X	X	X		X	X	X	X
156540	*MTAP*	4507	X	X		X			X	X	X	X
611427	*MTHFD1L*	25902	X	X	X	X	X	X		X	X	X

The search space sets refer to the gene sets created by the different SNP-to-gene methods explained in the text: 1** **Mbp, 1** **Mbp interval set; 0.5** **Mbp, 0.5** **Mbp interval set; 0.1** **Mbp, 0.1** **Mbp interval set; A, adjacent set; N, nearest set; R, resident set. The significance levels refer to the SNP stringency thresholds used: HS, highly significant; MHS, moderately high significant; MWS, moderately weak significant; WS, weakly significant. OMIM genes are the genes from the Online Mendelian Inheritance in Man database. WTCCC are the candidates from the Wellcome Trust Case Control Consortium study.

We also developed and tested two novel modules that search for genes that are targeted by common regulatory factors. These modules are based on the finding that disruption of regulatory elements in the genome that control gene expression levels can cause human diseases (Kleinjan and Coutinho 2009). Disruptions in these elements (cis-ruptions) could likely affect known disease genes, or novel genes with similar regulatory elements. As in the CPS module, significance of both the regulatory elements and the miRNA target genes are calculated through the standard one-tailed Fishers test and a *P*-value is assigned to each gene for prioritization.

The first of these modules, common regulatory targets (CRT), searches for genes in the implicated loci that bind transcription factors. Regulatory information was sourced from oRegAnno (Griffith et al. 2008), an experimentally derived and computationally predicted set of regulatory data. In *seeded* mode, we searched for common transcription factors that bind the regulatory elements of both the search genes and the known disease genes used as seeds. For the *ab initio* approach, CRT searches for enrichment of genes with common regulation among the loci in the gene search space.

A second regulatory module (MIR) looks for genes among the implicated loci that are common miRNA targets and in regulatory hubs. Dysfunction of miRNAs is believed to play a role in diseases of the heart, central nervous system, and immune system (Meola et al. 2009). MicroRNAs bind to mRNA, inhibiting protein synthesis through repression of translation or degradation of mRNA. Mutations in miRNAs or miRNA target sites prevent proper target recognition, leading to gene dysregulation. MicroRNA data were gathered from mirBase (Griffiths-Jones et al. 2008), a central online repository for miRNA nomenclature, sequence data, annotation, and target prediction. In *seeded* mode, MIR first searches for miRNAs that target the known disease genes. The remaining gene targets of these miRNAs are obtained from the database and searched for within the gene search space. In *ab initio* mode, this method searches for enriched miRNAs and returns the gene targets.

We then assessed *Gentrepid* predictions using the five modules on the GWAS-implicated loci relative to two validation sets (Table 1). The first set was the 13 OMIM known disease genes (known validation set). The second set was the five genes determined as candidates by the WTCCC study (WTCCC validation set). Finally, we studied novel predictions made by the *Gentrepid* modules and compared these to other GWAS and the current literature, where available.

## Results and Discussion

CAD is a chronic degenerative condition of the coronary arteries involving the build-up of atherosclerotic plaques, and a clinical presentation of myocardial infarction. CAD patients recruited by the WTCCC study had a validated history of either myocardial infarctions or coronary artery bypass surgery prior to the age of 66 years (Wellcome Trust Case Control Consortium 2007). For our analysis, we collated a set of 13 known CAD disease genes (Table 1) from OMIM. These relate to metabolism, transport, and signaling of low-density lipoproteins (LDL).

The original data from the WTCCC had one highly significant locus and six moderately associated loci. For the least significant SNP association level generated (WS), the data had 410 implicated loci, with ∼49% overlap with previously implicated regions from OMIM (Fig. 1). For each of the four SNP sets created (HS, MHS, MWS, and WS), six gene search spaces were generated (resident, nearest, adjacent, 0.1 Mbp, 0.5 Mbp, and 1 Mbp), totalling 24 search space sets. The largest gene search space was 2317 annotated genes (WS, 1 Mbp).

**Figure 2:**
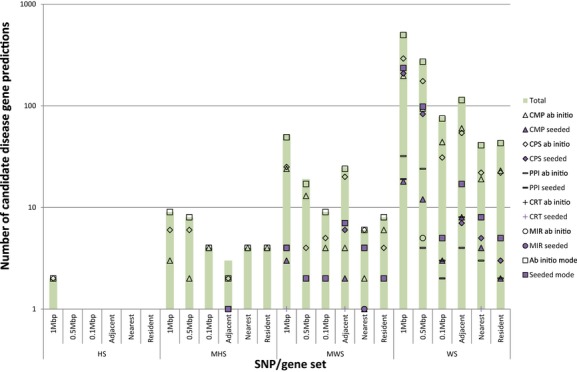
Number of significant predictions for CAD. The data are split across SNP/gene sets and are represented on a log10 scale. As per the key, the total predictions are shown by the purple bar, *seeded* mode predictions are the shapes in light grey with black border, *ab initio* predictions in white with black border. CMP predictions as triangles, CPS predictions as diamonds, PPI predictions as horizontal bars, CRT predictions as crosses, MIR predictions as circles, and the combined predictions as squares. The WS sets and 1 Mbp mappings had the most prediction results.

The number of predictions made by *Gentrepid* varied by search space, and was at most 525 genes for the WS 1 Mbp search space (Fig. 2). Breaking this down by module, *Gentrepid* CPS predicted up to 208 genes in *seeded* mode; and up to 292 genes in *ab initio* mode. CMP *seeded* predicted up to 18 genes and CMP *ab initio* mode predicted 197 genes. For PPI *seeded*, up to 39 genes interacted with the known seed genes, with 19 genes passing the significance test. PPI *ab initio* had over 1000 interacting genes with 32 genes passing the significance test. The regulatory modules had very few predictions; CRT *seeded* produced one gene prediction, MIR *seeded* predicted at most one candidate, while MIR *ab initio* generated at most five predictions. The top predictions are listed in Table 2 and the full list of significant predictions is in Table S1.

**Table 2:** Top CAD predictions made by *Gentrepid*.

Gene accession (OMIM)	Gene (HGNC)	Locus	Genetic support	Resident	Near	Adjacent	0.1 Mbp	0.5 Mbp	1 Mbp	Method	Common biological support	Score	Rank
601470	***CX3CR1***	**3p22.2**	✓✓			✓		✓	✓	CPS-s	Cytokine–cytokine receptor interaction	◊◊◊	2
147545	***IRS1***	**2q36.3**	✓✓		✓	✓			✓	CPS-s	Insulin signaling	◊	1
152200	***LPA***	**6q26**	✓					✓	✓	CMP-ab	DUF1986|Kringle| Trypsin	○○○○○	53
163729	***NOS3***	**7q36.1**	✓					✓	✓	CPS-s	Metabolic pathways	◊◊◊◊	1
173510	***CD36***	**7q21.11**	✓✓			✓		✓	✓	CPS-s	Phagosome	◊	3
605009	*ADAMTS7*	15q25.1	✓✓	✓	✓	✓	✓	✓	✓	CMP-ab	ADAM_spacer1| Pep_M12B_propep| Reprolysin|TSP_1	○○○○○	1
600160	*CDKN2A*	9p21.3	✓✓✓✓				✓	✓	✓	CPS-ab	Non–small cell lung cancer	◊	6
600431	*CDKN2B*	9p21.3	✓✓✓✓			✓	✓	✓	✓	CPS-ab	Pathways in cancer	◊◊◊	2
156540	*MTAP*	9p21.3	✓✓✓✓					✓	✓	CPS-s	Metabolic pathways	◊◊◊◊	1
611427	*MTHFD1L*	6q25.1	✓✓✓	✓	✓	✓	✓	✓	✓	CPS-s	Metabolic pathways	◊◊◊◊	1
138352	*GRIN2B*	**12p13.1**	✓		✓	✓		✓	✓	PPI-s	*IRS1*	◊◊◊	4
600297	*CDX2*	**13q12.2**	✓					✓	✓	MIR-ab	hsa-mir-181b-1 (MI0000270)	◊◊	1
603722	*IKBKAP*	**9q31.3**	✓	✓	✓	✓	✓	✓	✓	CPS-ab	CD40L Signaling Pathway	◊◊	7
176541	*TYK2*	**19p13.2**	✓						✓	CPS-ab	Jak-STAT signaling pathway	◊	10
601153	*FHIT*	**3p14.2**	✓	✓	✓	✓	✓	✓	✓	CPS-ab	Small cell lung cancer	◊◊◊	4
135630	*ITGB1*	10p11.22	✓						✓	PPI-s	*CD36*	◊◊◊◊	9
173470	*ITGB3*	17q21.32	✓						✓	PPI-s	*CD36*	◊◊◊	14
600065	*ITGB2*	21q22.3	✓✓					✓	✓	CPS-s	Phagosome	◊	3
147561	*ITGB5*	3q21.2	✓✓✓						✓	CMP-ab	EGF_2|Integrin_B_tail| Integrin_b_cyt| Integrin_beta	○○○○○	7
147557	*ITGB4*	17q25.1	✓✓					✓	✓	CMP-ab	EGF_2| Integrin_B_tail| Integrin_beta	○○○○○	15
125855	*DGKA*	12q13.2	✓✓						✓	CPS-s	Metabolic pathways	◊◊◊◊	1
604070	*DGKB*	7p21.2	✓	✓	✓	✓	✓	✓	✓	CPS-s	Metabolic pathways	◊◊◊◊	1
604071	*DGKH*	13q14.11	✓						✓	CPS-s	Metabolic pathways	◊◊◊◊	1
607021	*SEZ6L*	22q12.1	✓✓✓	✓	✓	✓	✓	✓	✓	CMP-ab	CUB|Sushi	○○○○○	1
608398	*CSMD2*	1p34.3	✓✓✓	✓	✓	✓	✓	✓	✓	CMP-ab	CUB|Sushi	○○○○○	1
601692	*TGFBI*	5q31.2	✓		✓	✓	✓	✓	✓	CMP-ab	Fasciclin	○○○○	30
608777	*POSTN*	13q13.3	✓		✓	✓	✓	✓	✓	CMP-ab	Fasciclin	○○○○	30
600797	*IRS2*	13q34	✓✓		✓	✓		✓	✓	CMP-s	IRS|PH	✓✓✓	1
173350	*PLG*	6q26	✓						✓	CMP-s	DUF1986|Kringle| Trypsin	✓✓✓✓	1
–	*LRP11*	6q25.1	✓	✓	✓	✓	✓	✓	✓	CMP-s	Ldl_recept_a	✓✓	1
120280	*COL11A1*	1p21.1	✓	✓	✓	✓	✓	✓	✓	CPS-s	ECM–receptor interaction	◊	1
120090	*COL4A2*	13q34	✓✓	✓	✓	✓	✓	✓	✓	CPS-s	ECM–receptor interaction	◊	1
600514	*RELN*	7q22.1	✓	✓	✓	✓	✓	✓	✓	CPS-s	ECM–receptor interaction	◊	1
605264	*SORBS1*	10q23.33	✓✓	✓	✓	✓	✓	✓	✓	CPS-s	Insulin signaling	◊	1
138550	*PYGB*	20p11.21	✓✓	✓	✓	✓	✓	✓	✓	CPS-s	Insulin signaling	◊	1
603961	*SEMA3A*	**7q21.11**	✓		✓	✓	✓	✓	✓	CPS-ab	Axon guidance	◊◊◊	1
611766	*MTFMT*	15q22.31	✓✓				✓	✓	✓	CPS-ab	One carbon pool by folate	◊◊◊	1
108355	*GRB2*	17q25.1	✓			✓	✓	✓	✓	PPI-s	*IRS1*	◊◊◊◊	1
612375	*AIDA*	1q41	✓			✓	✓	✓	✓	MIR-ab	hsa-mir-181b-1 (MI0000270)	◊◊	1
601656	*GATA6*	18q11.2	✓			✓		✓	✓	MIR-ab	hsa-mir-181b-1 (MI0000270)	◊◊	1

Genes and loci in bold have been previously associated with the disease. Genes underlined are the WTCCC candidates. Key to genetic support column: HS, ✓✓✓✓; MHS, ✓✓✓; MWS, ✓✓; WS, ✓. Method: ab, *ab initio*; s, *seeded*. Common biological support column depends on method. For CMP-s, common gene and common domain are listed. For CMP-ab, only the common domain. For CPS-s and CPS-ab, the common pathway is listed. For PPI-s, the HGNC gene name of the gene(s) are listed. For MIR-s, the common miRNA ID is listed. For CRT, the common oRegAnno ID is listed. *Gentrepid* scoring: CMP-ab: ○○○○○, log χ^2^ ≥ 2.5; ○○○○, 2 ≤ log *χ*^2^ < 2.5; ○○○, 1.5 ≤ log *χ*^2^ < 2; ○○, 1 ≤ log *χ*^2^ < 1.5; ○, log *χ*^2^ < 1. CMP-s: ✓✓✓✓, Sc > 0.7; ✓✓✓, Sc > 0.6; ✓✓, Sc > 0.5; ✓, Sc > 0.4. Other: ◊◊◊◊, *P *< 0.005; ◊◊◊, *P *< 0.01; ◊◊, *P *< 0.025; ◊, *P *< 0.05. Rank represents ranking score in prioritization of gene in specific set and search space and module, not overall ranking.

### Candidate predictions in previously implicated loci

Our first assessment of the *Gentrepid* predictions tested its ability to predict known disease genes and the disease gene candidates from previous studies. We looked for the predictions and ranks of known disease genes implicated in CAD from the OMIM database, and for the candidate disease genes implicated by the WTCCC study (Table 1).

Of the 13 OMIM disease genes implicated in CAD, up to six were in at least one of the CAD gene search spaces constructed from the WTCCC SNPs and seven were outside the search spaces and discarded from the validation process. Four of these were detected by CPS from pathways using the *seeded* or *ab initio* method. Three of these genes were supported by multiple SNPs in the GWAS data. These were chemokine (C-X3-C motif) receptor 1 *CX3CR1* (MIM 601470) in 3p22.1-3, a chemokine involved in LDL signaling pathways; and *CD36* (MIM 173510) and insulin receptor substrate 1 *IRS1* (MIM 147545), which are both receptors in the adipocytokine signaling pathway. A fourth gene, nitric oxide synthase 3, *NOS3* (MIM 163729) in 7q21.11, though only supported by SNPs in the WS set, was predicted through CPS *seeded*. Lipoprotein A (*LPA*, 6q27, MIM 152200), also only supported by weak SNP association signals, was predicted through CMP (Table 2).

Of the five reported WTCCC genes of interest, all five genes were predicted by *Gentrepid* by either CPS or CMP (Table 2) and were highly ranked. Cyclin-dependent kinases inhibitors *CDKN2A*/*B* (MIM 600160/MIM 600431) and a phosphorylase *MTAP* (MIM 156540), associated with a single highly significant locus (9p21), were predicted via common metabolic pathways along with a modestly associated dehydrogenase *MTHFD1L* (6q25, MIM 611427). The metalloproteinase *ADAMTS7* (MIM 605009), implicated by a modest association (15q24), had common domains with another metalloproteinase in the gene search spaces.

### Novel candidate disease gene predictions

Our next assessment was to analyze the candidate disease gene predictions that had not been previously reported at the time of the generation of the data. First, we looked at the candidates predicted in loci that were previously implicated but had no known or candidate disease gene, as not all the loci listed in OMIM have candidates. Of 15 previously determined disease loci, all contained WS SNPs. Only five loci contained MWS SNPs and one locus contained MHS SNPs. None of the previously implicated loci were detected at the HS level. The largest numbers of SNPs were associated with 2q36.3 and 3p22-p21 in which *CX3CR1* (MIM 601470) and *IRS1* (MIM 147545) have previously been identified as the disease genes. Predictions within previously determined loci for which a known gene has not been determined were based on very weak genetic signals, typically one, or at most two WS SNPs. They include tyrosine kinase 2 *TYK2* (MIM 176541) in 19q13, regulatory factor X *RFX5* (MIM 601863) in 1q21.2, fragile histidine triad *FHIT* (MIM 601153) in 3p14.2, and the tumor necrosis factor receptors *TNFRSF10A-D* (MIM 603611, MIM 603612, MIM 603613, MIM 603614) in 8p21.2.

In addition, 15 alternative gene predictions were made for loci with previously determined disease genes. Of these, the IκB kinase complex-associated protein *IKBKAP* (MIM 603722) in 9q31.2 was recently shown to be differentially regulated in patients with acute myocardial infarction compared to controls (Dabek et al. 2009). The disease gene previously implicated for this region is the ATP-binding cassette *ABCA1* (MIM 600046).

### Common pathway candidates

We then looked at the novel candidates from each of the modules for the regions implicated by our novel methodology and SNP/gene mappings. Two of the most significant pathways predicted by *Gentrepid* CPS in loci novel to the WTCCC study were diabetes related. In the “Type II diabetes mellitus” pathway (MWS set, *P*_path,adjacent_ = 0.005), three genes in three novel loci (10q23, 13q34, 20p11) were implicated in addition to the known disease gene *IRS1* (MIM 147545). In the MWS set, the “Insulin signaling pathway” was the most significant (*P*_path_,_nearest_ = 0.0003). Patients with T2D are known to have a higher risk of CAD. The possible commonality of pathways underlying CAD and T2D was raised by Torkamani et al. (2008) based on their analysis of the WTCCC data. In addition two hypoxia-related pathways suggested hypoxia-inducible factor *HIF1A* (MIM 603348) as a candidate. Overall, 56 novel pathways were predicted in *ab initio* mode across all the gene search spaces (Table S2).

We also wished to compare our pathway results to predictions from the Framingham study (de las Fuentes et al. 2012) which used variable set enrichment analysis (VSEA) to uncover significant pathways. Of the 25 pathways they found to be significant, the “Rac 1 cell motility signaling pathway” was the only significant pathway in both our studies. Of the 18 genes predicted by this pathway in the Framingham study, CPS *ab initio* and *seeded* module (Table S1) predicted three of those genes: *RAC1* (Ras-related C3 botulinum toxin substrate 1, MIM 602048), *PDGFRA* (Alpha-type platelet-derived growth factor receptor, MIM 173490), and *PLD1* (phospholipase D1, phosphatidylcholine-specific, MIM 602382) (*P*_WS,adjacent_ = 0.007). *WASF1* (Wiskott-Aldrich syndrome protein family member 1, MIM 605035), and the enzyme LIM domain kinase (*LIMK1*, MIM 601329, *P*_WS,0.5 Mbp_ = 0.018) were not in the Framingham study but were predicted by our method. *LIMK1* (MIM 601329) may be involved in cardiovascular disease through its interactions with BMP type II receptor (*BMPR2*, MIM 600799) (Scott and Olson 2007).

### Domain homology candidates

Protein domains are highly conserved globular structures each with their own biochemical function (Finn et al. 2008). In combination, domains can be used to assign a function to a protein and its gene (Patthy 2003). Thus using domain homology, genes with unknown functions can potentially be identified as candidates. *Gentrepid* CMP *seeded* found five genes with similarities to LDL receptor-like protein *LRP6* (MIM 603507) in the gene search spaces; two genes homologous to the lipoprotein carrier *LPA* (MIM 152200): *PLG* (MIM 173350) and *LPAL2* (MIM 611682); and a matrix metalloproteinase (*MMP15*, MIM 602261) similar to *MMP3* (MIM 185250), involved in extracellular matrix (ECM) breakdown (Table S3). Many plausible candidates were predicted by CMP *ab initio* (Table S4). Cell–cell and ECM adhesion, as well as their remodeling, featured prominently. Genes with the strongest genetic support are the vascular adhesion factors *SEZ6L* (22q11.23, MIM 607021) and *CSMD2* (1p35.1, MIM 608398). Adhesion between the cell and the ECM is implicated by multiple integrins and matrix metalloproteases as well as by transforming growth factor *TGFBI* (MIM 601692) and periostin *POSTN* (MIM 608777). *TGFBI* (MIM 601692) binds to type I, II, and IV collagens. Other adhesion genes predicted were adhesion G-protein coupled receptors. Lipid signaling was also implicated by phospholipases, DAG kinases, and protein kinase C-like genes (Table S4).

### Predictions from the PPI module

**Figure 3:**
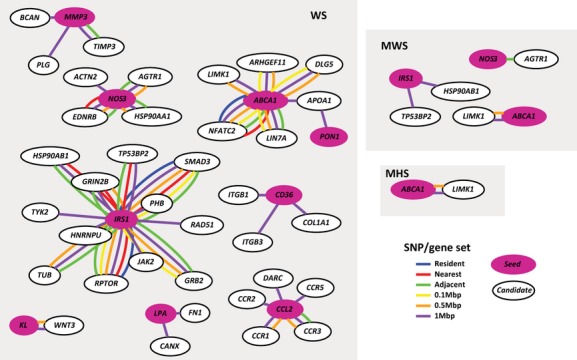
CAD PPI *seeded* interactions. The genes in magenta are the known OMIM seed genes used for the PPI module. The lines represent an interaction. The different colors represent the gene search space the interaction arises from. Resident set interactions in blue, nearest set in red, adjacent set in green, 0.1 Mbp set in yellow, 0.5 Mbp set in orange, and 1 Mbp set in purple.

For the PPI module, where the search was limited to direct interaction partners, the sets with more genes (1 and 0.5 Mbp) and less stringent significant thresholds (WS and MWS) had the greatest number of predictions in both *seeded* and *ab initio* modes. Some of the predictions were the same as those from the CPS and CMP modules, such as the chemokine receptors *CCR1, 2, 3*, and *5* (MIM 601159, MIM 601267, MIM 601268, and MIM 601373) that interact with known disease gene *CCL2* (MIM 158105), and *LIMK1* (MIM 601329) that interacts with *ABCA1* (MIM 600046) (Fig. 3 and Table S5).

**Figure 4:**
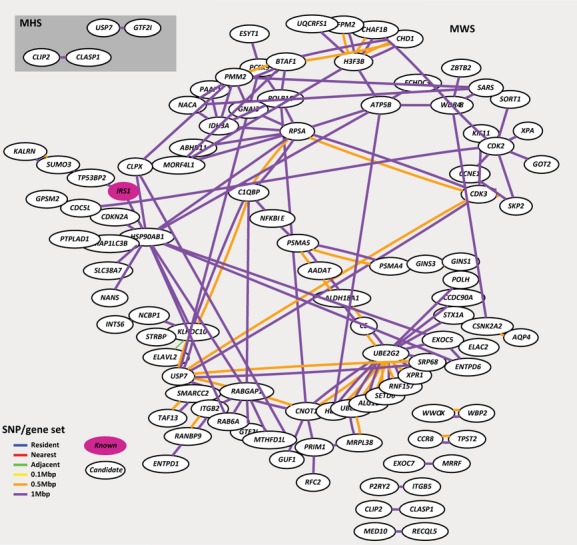
CAD PPI *ab initio* interactions for the MHS and MWS sets. The genes in magenta are the known OMIM seed genes used for the PPI module. The lines represent an interaction. The different colors represent the gene search space the interaction arises from. Resident set interactions in blue, nearest set in red, adjacent set in green, 0.1 Mbp set in yellow, 0.5 Mbp set in orange, and 1 Mbp set in purple.

The significant predictions of the PPI *ab initio* module are listed in Tables S6, S7. Visualizations of the interactions (Fig. 4) implicate protein interaction hubs such as the ubiquitin-conjugating enzyme *UBE2G2* (MIM 603124) or the ubiquitin-specific processing protease *USP7* (MIM 602519). The ubiquitin–proteasome complex and proper protein degradation are involved in cardiovascular physiology and disease with roles in endothelial function and atherosclerosis (Depre et al. 2010).

### Predictions from regulatory modules

Very few predictions were returned for the *Gentrepid* regulatory modules MIR and CRT. For CRT, the known *IRS1* (MIM 147545) gene was the sole prediction. For the MIR module, *IRS1* (MIM 147545) was the only significant prediction in *seeded* mode. The miRNAs that implicated this gene were mir-126, known to be involved in angiogenesis (Van Solingen et al. 2009) and mir-145, involved in vascular smooth muscle differentiation (Cordes et al. 2009) and possibly CAD (Fichtlscherer et al. 2010). In *ab initio* mode, several genes were predicted from the WS sets which were all implicated by the family of miRNAs from the mir-181 precursor (Table S8). The homeobox *CDX2* (MIM 600297), transcription factor *GATA6* (MIM 601656) and axin interactor *AIDA* (MIM 612375) are regulated by the mir-181 family. Mir-181 target genes are involved in myogenesis, muscle regeneration (Naguibneva et al. 2006), and hematopoiesis (Chen et al. 2004). *GATA6* (MIM 601656) mutations are known to cause congenital heart defects (Maitra et al. 2009). Thus risk alleles of this transcription factor could plausibly contribute to CAD. *AIDA* (MIM 612375) is highly expressed in the heart and skeletal muscle, also making it an interesting candidate (Rui et al. 2007). Predictions of a read-through transcript *TGIF2-C20orf24* and DNA methyltransferase *DNMT1* (MIM 126375) by their common regulator mir-148 are interesting predictions, as mir-148a also promotes skeletal muscle differentiation (Zhang et al. 2012).

### Further case study: CAD meta-analyses

A recent meta-analysis GWA on CAD called the CARDIoGRAMplusC4D study (Deloukas et al. 2012) discovered 15 novel association loci and listed 20 likely candidate genes. We again wished to compare our method to this study's results. First, we checked if their significant loci were a subset of the less significant loci from the WTCCC and found very few overlaps. Whether these overlaps are simply due to chance or a significant association that was missed in the stringent threshold is hard to determine. Nonetheless, we still wished to see if our method was capable of selecting appropriate candidates from this new set of loci. We then took the novel loci, mapped them to their adjacent genes and ran *Gentrepid*, comparing their candidates with our predictions. Of the 20 genes that were selected as candidates from the CARDIoGRAMplusC4D study, 16 genes were mapped by our adjacent SNP-to-gene mapping, and *Gentrepid* predicts and prioritizes 11 of these genes (Table 3 and Table S9). *Gentrepid* also made three alternate predictions: the leucine-rich PPR motif-containing protein, mitochondrial gene (*LRPPRC*, MIM 607544), mitogen-activated protein kinase kinase kinase 4 (*MAP3K4*, MIM 602425) and guanylate cyclase soluble subunit beta-1 (*GUCY1B3*, MIM 139397). *GUCY1B3* (MIM 139397) is known to interact with the endothelial NOS (*NOS3*, MIM 163729), a gene associated with ischemic heart disease and hypertension (Casas et al. 2004). Our analyses once again showed that the system was capable of making valid predictions based on biological knowledge, and also generate novel hypotheses based on enrichment of common pathways and functional domains within the data.

**Table 3:** CAD predictions made by *Gentrepid* for the CARDIoGRAMplusC4D loci.

Gene accession (OMIM)	Gene (HGNC)	CARDIoGRAMplusC4D SNP	Method	Common biological support	Score	Rank
190030	*FES*	rs17514846	CMP-ab	Pkinase_tyr	○	2
165070	*FLT1*	rs9319428	CMP-ab	Pkinase_tyr	○	2
193002	*SLC18A1*	rs264	CMP-ab	MFS_1	○	1
604190	*SLC22A4*	rs273909	CMP-ab	MFS_1	○	1
131243	*EDNRA*	rs1878406	CMP-s	7tm_1	✓	3
173350	*PLG*	rs4252120	CMP-s	Kringle	✓✓✓✓	1
605460	*ABCG8*	rs6544713	CMP-s	ABC_tran	✓	2
147880	*IL6R*	rs4845625	CPS-ab	IL 6 signaling pathway| Role of ERBB2 in signal transduction and oncology	◊◊◊	1
607544	***LRPPRC***	rs6544713	CPS-ab	IL 6 signaling pathway| Role of ERBB2 in signal transduction and oncology	◊◊◊	1
605459	*ABCG5*	rs6544713	CPS-s	Nuclear receptors in lipid metabolism and toxicity	^*^	2
139396	*GUCY1A3*	rs7692387	CPS-s	Long-term depression	^*^	6
139397	***GUCY1B3***	rs7692387	CPS-s	Long-term depression	^*^	6
609708	*LPL*	rs264	CPS-s	Low-density lipoprotein (LDL) pathway during atherogenesis	◊◊	1
602425	***MAP3K4***	rs4252120	CPS-s	MAPKinase Signaling Pathway	^*^	8

Method: ab, *ab initio*; s, *seeded*. Common biological support column depends on method. For CMP-s, common gene and common domain are listed. For CMP-ab, only the common domain. For CPS-s and CPS-ab, the common pathway is listed. For PPI-s, the HGNC gene name of the gene(s) are listed. For MIR-s, the common miRNA ID is listed. For CRT, the common oRegAnno ID is listed. *Gentrepid* scoring: CMP-ab: ○○○○○, log χ^2^ ≥ 2.5; ○○○○, 2 ≤ log *χ*^2^ < 2.5; ○○○, 1.5 ≤ log *χ*^2^ < 2; ○○, 1 ≤ log *χ*^2^ < 1.5; ○, log *χ*^2^ < 1. CMP-s: ✓✓✓✓, Sc > 0.7; ✓✓✓, Sc > 0.6; ✓✓, Sc > 0.5; ✓, Sc > 0.4. Other: ◊◊◊◊, *P *< 0.005; ◊◊◊, *P *< 0.01; ◊◊, *P *< 0.025; ◊, *P *< 0.05; ^*^, not significant. Rank represents ranking score in prioritization of gene in module, not overall ranking. Genes in bold are candidate predictions not selected by the CARDIoGRAMplusC4D.

### Caveats

Although we aimed to predict and prioritize a list of candidate disease genes, there still remains a reasonably high probability (50/50 at worst) that a result is a false positive. From our methods paper (Ballouz et al. 2011), we calculated the specificity of the system to be between 0.55 and 1, depending on the method used. Further to this, even the list of candidates used to validate our study may also be false positives, and therefore skew our calculations. For instance, the 6q25 locus was not replicated in other studies (Kathiresan et al. 2009) and therefore the *MTHFD1L* (MIM 611427) gene may be a false-positive result. However, the OMIM validation set contained genes that were selected by our system, thereby validating the technique to some extent. Also, by demonstrating that *Gentrepid* selected at least half of the candidates from the CARDIoGRAMplusC4D, the system appears competitive with meta-analysis methods used by researchers to determine candidates. The system also made alternate predictions which might be of interest too. Another point to note is the low concordance of the pathway enrichment results with the Framingham study (de las Fuentes et al. 2012). A few reasons for this include the fact that we used different gene data sets as input to which the methods are highly sensitive to (Glaab et al. 2012). A very highly annotated gene in one set yet missing in the other will skew results depending how the significance is calculated. Although it would have been reassuring to have obtained a larger overlap in the pathways, it nonetheless brings to light how dependent the methods, in particular gene set enrichment, are on the underlying data.

## Conclusions

We performed an extensive analysis of the GWAS data for CAD. The approach used four sets of significant SNPs filtered at different significance thresholds. Gene search spaces were generated in six different ways and the resulting sets analyzed with the *Gentrepid* candidate gene prediction system. The results show that using a less stringent significance threshold increases the noise in the system, yet even so brings out likely candidate disease genes. Looking beyond the nearest gene in order to find suitable disease candidates, in both the adjacent and BY approaches, is also valuable for the analysis of GWAS data, in particular for the gene desert regions with regulatory elements.

### Which method is best?

Biological filtering improves the amount of knowledge extracted from the study. Using the blind method (*ab initio*), no prior disease gene information is required, allowing for the discovery of novel pathways and regulatory elements that may be important in the disease that were not previously considered, along with protein domains attributing novel functions to the mechanisms behind the disease. The few functionally annotated miRNAs that produced predictions had relevant biological functionality in the phenotype and it would be very interesting to prod further into these, in particular genes *GATA6* (MIM 601656) and *AIDA* (MIM 612375). MicroRNA research is expanding and will produce motivating hypotheses relevant to disease. Further to this, genes such as *RAC1* (MIM 602048)*, LIMK1* (MIM 601329), *SEZ6L* (MIM 607021), and *CSMD2* (MIM 608398) also warrant further investigation as they were predicted by multiple methods or had strong genetic support.

Generally, we recommend the use of the adjacent gene method or genes within a 0.1–0.5 Mbp interval to create the search space sets. Most systems use biological pathways and GO terms to predict disease gene candidacy (Tranchevent et al. 2011), therefore the use of CMP would be recommended as it is unique to *Gentrepid*.

Although there are alternate methods that capitalize on gene and pathway enrichment analysis (Raychaudhuri et al. 2009; Duncan et al. 2010; de las Fuentes et al. 2012) and PPI data (Jensen et al. 2011), our method incorporates multiple methods along with functional protein domain information. The *Gentrepid* webserver is available for free usage by educational and nonprofit research institutes (https://www.gentrepid.org). Registration is free and data are stored remotely and securely. Each of the methods highlighted here can be performed with the exception of the regulatory modules CRT and MIR under development. The only input required is a list of SNPs or markers and an optional phenotype for the *seeded* mode.

As in all candidate disease gene methods, it is still difficult to perform a fair assessment of the results without further biochemical functional studies. Overall, we believe our pipeline is a suitable methodology for generating plausible hypotheses from GWAS. The study demonstrates that using existing knowledge and a holistic multiple loci approach provides insight into what is a very complex disease.

## Supporting Information

**Figure S1**. Workflow diagram. SNPs are filtered for significance at different thresholds. These SNPs are then naively clustered if in close proximity (within 50 Kbp). SNP loci are then mapped to genes in one of six ways, creating six gene search space sets for each significance threshold. Each gene search space is then used as input into *Gentrepid*, with bioinformatic tests performed, resulting in a list of prioritized disease candidate genes. **Table S1**. *Gentrepid* gene predictions for the CAD phenotype. **Table S2**. Significantly enriched pathways for the CAD phenotype. **Table S3**. CAD CMP *seeded* results. **Table S4**. CAD CMP *ab initio* results. **Table S5**. CAD PPI *seeded* interactions, with scores and ranks. **Table S6**. CAD PPI *ab initio* significant interactions and predictions for the MHS and MWS sets. **Table S7**. CAD PPI *ab initio* interactions for the WS sets. **Table S8**. CRT and MIR results for the CAD phenotype. **Table S9**. CARDIoGRAMplusC4D study loci and candidate genes.
