# Supplementary material for: Candidate disease gene prediction using Gentrepid: application to a genome-wide association study on coronary artery disease
Source: Mol Genet Genomic Med. 2013 Nov 13;2(1):44–57. doi: 10.1002/mgg3.40 (PMC3907915; doi:10.1002/mgg3.40)
Supplement: Supplementary file 1 — Figure S1. Workflow diagram. SNPs are filtered for significance at different thresholds. These SNPs are then naively clustered if in close proximity (within 50 Kbp). SNP loci are then mapped to genes in one of six ways, creating six gene search space sets for each significance threshold. Each gene search space is then used as input into Gentrepid, with bioinformatic tests performed, resulting in a list of prioritized disease candidate genes. Table S1. Gentrepid gene predictions for the CAD phenotype. Table S2. Significantly enriched pathways for the CAD phenotype. Table S3. CAD CMP seeded results. Table S4. CAD CMP ab initio results. Table S5. CAD PPI seeded interactions, with scores and ranks. Table S6. CAD PPI ab initio significant interactions and predictions for the MHS and MWS sets. Table S7. CAD PPI ab initio interactions for the WS sets. Table S8. CRT and MIR results for the CAD phenotype. Table S9. CARDIoGRAMplusC4D study loci and candidate genes. [file mgg30002-0044-sd1.pdf]

Supporting Information

Supp. Figure S1. Workflow diagram.. 2
Supp. Table S1. *Gentrepid* gene predictions for the CAD phenotype 3
Supp. Table S2. Significantly enriched pathways for the CAD phenotype 14
Supp. Table S3. CAD CMP *seeded* results 17
Supp. Table S4. CAD CMP *ab initio* results 18
Supp. Table S5. CAD PPI seeded interactions, with scores and ranks 21
Supp. Table S6. CAD PPI *ab initio* significant interactions and predictions for the MHS and MWS sets 23
Supp. Table S7. CAD PPI *ab initio* interactions for the WS sets 24
Supp. Table S8. CRT and MIR results for the CAD phenotype 26
Supp. Table S9. CARDIoGRAMplusC4D study loci and candidate genes 26

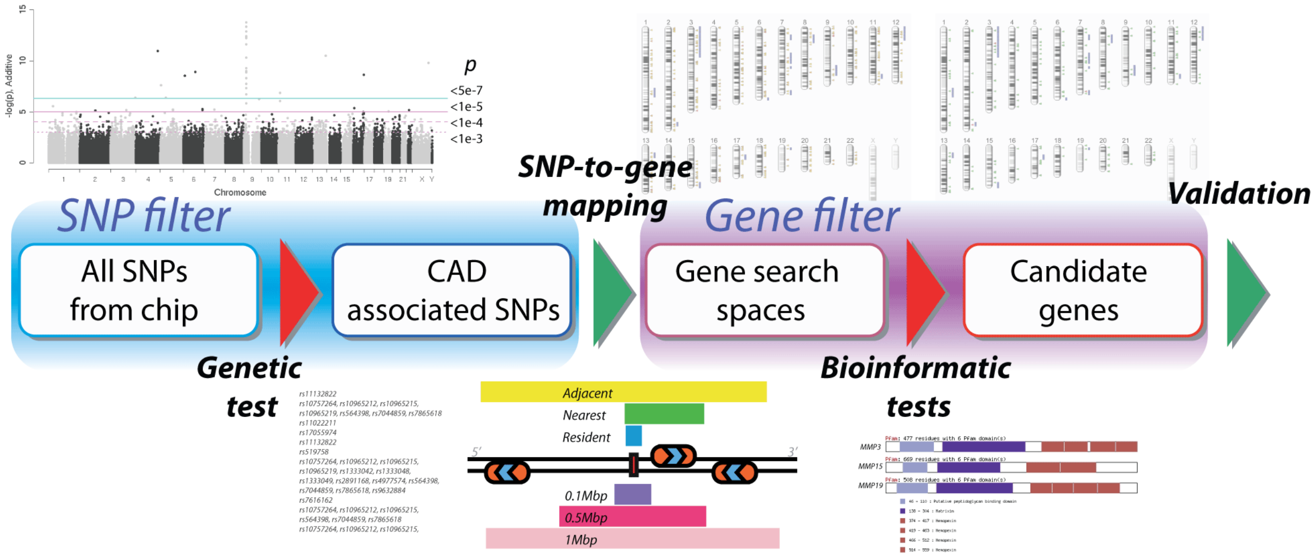

Supp. Figure S1. Workflow diagram. SNPs are filtered for significance at different thresholds. These SNPs are then naïvely clustered if in close proximity (within 50Kbp). SNP loci are then mapped to genes in 1 of 6 ways, creating 6 gene search space sets for each significance threshold. Each gene search space is then used as input into *Gentrepid*, with bioinformatic tests performed, resulting in a list of prioritised disease candidate genes.

Supp. Table S1. *Gentrepid* gene predictions for the CAD phenotype

Gene	Locus	Genetic support	Resident	Near	Adjacent	0.1Mbp	0.5Mbp	1Mbp	Method	Common biological support	Score	Rank
ENO1	1p36.23	●			✔		✔	✔	CPS-s	Metabolic pathways	◊◊◊◊	1
RERE	1p36.23	●					✔	✔	PPI-ab	11143, 6613, 2202, 10346, 7994	◊	48
H6PD	1p36.22	●			✔		✔	✔	CPS-s	Metabolic pathways	◊◊◊◊	1
OPRD1	1p35.3	●			✔	✔	✔	✔	CMP-ab	7TM_GPCR_Srsx| 7TM_GPCR_Srv| 7TM_GPCR_Srx| 7tm_1	○○○○○	8
RAB42	1p35.3	●						✔	CMP-ab	Arf| Miro| Ras	○○○○○	55
EPB41	1p35.3	●		✔	✔	✔	✔	✔	CMP-ab	FA| FERM_C| FERM_M| FERM_N	○○○○○	1
PTPRU	1p35.3	●						✔	CMP-ab	MAM| Y_phosphatase| fn3	○○○○○	66
MECR	1p35.3	●						✔	CPS-s	Metabolic pathways	◊◊◊◊	1
CSMD2	1p34.3	●●●	✔	✔	✔	✔	✔	✔	CMP-ab	CUB| Sushi	○○○○○	1
DOCK7	1p31.3	●			✔	✔	✔	✔	CMP-ab	DUF3398| Ded_cyto	○○○○○	29
PTGFR	1p31.1	●						✔	CMP-ab	7TM_GPCR_Srv| 7TM_GPCR_Srx| 7tm_1	○○○○○	57
NEGR1	1p31.1	●			✔				CMP-ab	I-sset| V-set| ig	○○○○○	28
FPGT	1p31.1	●				✔	✔	✔	CPS-s	Metabolic pathways	◊◊◊◊	1
HFM1	1p22.2	●			✔	✔	✔	✔	CMP-ab	DEAD| Helicase_C| ResIII	○○○○○	21
CDC7	1p22.2	●			✔		✔	✔	PPI-ab	5001, 1017, 10963, 4174, 1029, 1018, 84078, 56902, 51082, 5708, 84940, 5707, 51719	◊	59
COL11A1	1p21.1	●	✔	✔	✔	✔	✔	✔	CPS-s	ECM-receptor interaction	◊	1
SORT1	1p13.3	●●				✔	✔	✔	CMP-ab	BNR	○○○○	39
CELSR2	1p13.3	●●	✔	✔	✔	✔	✔	✔	CMP-ab	EGF| Laminin_G_1| Laminin_G_2	○○○○○	3
PSMA5	1p13.3	●●					✔	✔	CMP-ab	Proteasome| Proteasome_A_N	○○○○○	6
AMPD2	1p13.3	●●						✔	CPS-s	Metabolic pathways	◊◊◊◊	1
SARS	1p13.3	●●				✔	✔	✔	CPS-ab	Aminoacyl-tRNA biosynthesis	◊◊	2
GSTM4	1p13.3	●●						✔	CPS-ab	Drug metabolism - cytochrome P450	◊◊	7
GSTM5	1p13.3	●●						✔	CPS-ab	Drug metabolism - cytochrome P450	◊◊	7
GSTM3	1p13.3	●●						✔	CPS-ab	Drug metabolism - cytochrome P450	◊◊	7
GSTM2	1p13.3	●●						✔	CPS-ab	Drug metabolism - cytochrome P450	◊◊	7
GSTM1	1p13.3	●●						✔	CPS-ab	Drug metabolism - cytochrome P450	◊◊	7
WDR47	1p13.3	●●					✔	✔	PPI-ab	6919, 55743	◊	166
NRAS	1p13.2	●						✔	CMP-ab	Arf| GTP_EFTU| Miro| Ras	○○○○○	30
DENND2C	1p13.2	●						✔	CMP-ab	DENN| dDENN| uDENN	○○○○○	35
AMPD1	1p13.2	●						✔	CPS-s	Metabolic pathways	◊◊◊◊	1
ADAM30	1p12	●						✔	CMP-ab	Disintegrin| Pep_M12B_propep| Reprolysin	○○○○○	52
NOTCH2	1p12	●						✔	CMP-ab	EGF| EGF_2| EGF_CA	○○○○○	58
HSD3B1	1p12	●			✔	✔	✔	✔	CPS-s	Metabolic pathways	◊◊◊◊	1
HSD3B2	1p12	●					✔	✔	CPS-s	Metabolic pathways	◊◊◊◊	1
PHGDH	1p12	●					✔	✔	CPS-s	Metabolic pathways	◊◊◊◊	1
HAO2	1p12	●					✔	✔	CPS-s	Metabolic pathways	◊◊◊◊	1
MAN1A2	1p12	●					✔	✔	CPS-s	Metabolic pathways	◊◊◊◊	1
HMGCS2	1p12	●						✔	CPS-s	Metabolic pathways	◊◊◊◊	1
										**1q21-q23**		
PI4KB	**1q21.2**	●						✔	CPS-s	Metabolic pathways	◊◊◊◊	1
RFX5	**1q21.2**	●						✔	CPS-ab	Tuberculosis	◊◊◊	5
OAZ3	**1q21.3**	●			✔	✔	✔	✔	CMP-ab	ODC_AZ	○○○○	39
BCAN	**1q23.1**	●						✔	CMP-ab	EGF| Lectin_C| Sushi| V-set| Xlink	○○○○○	10
INSRR	**1q23.1**	●					✔	✔	CMP-ab	Furin-like| Pkinase| Pkinase_Tyr| Recep_L_domain	○○○○○	12
OR6N2	**1q23.1**	●			✔	✔	✔	✔	CPS-ab	Olfactory transduction	◊◊◊	1
OR6K6	**1q23.1**	●			✔	✔	✔	✔	CPS-ab	Olfactory transduction	◊◊◊	1
OR6N1	**1q23.1**	●				✔	✔	✔	CPS-ab	Olfactory transduction	◊	5
OR10R2	**1q23.1**	●						✔	CPS-ab	Olfactory transduction	◊◊◊◊	2
OR10K1	**1q23.1**	●						✔	CPS-ab	Olfactory transduction	◊◊◊◊	2
OR10T2	**1q23.1**	●						✔	CPS-ab	Olfactory transduction	◊◊◊◊	2
OR10K2	**1q23.1**	●						✔	CPS-ab	Olfactory transduction	◊◊◊◊	2
OR10X1	**1q23.1**	●					✔	✔	CPS-ab	Olfactory transduction	◊◊◊◊	1
OR6K2	**1q23.1**	●					✔	✔	CPS-ab	Olfactory transduction	◊◊◊◊	1
OR6K3	**1q23.1**	●					✔	✔	CPS-ab	Olfactory transduction	◊◊◊◊	1
OR6Y1	**1q23.1**	●					✔	✔	CPS-ab	Olfactory transduction	◊◊◊◊	1
OR10Z1	**1q23.1**	●					✔	✔	CPS-ab	Olfactory transduction	◊◊◊◊	1
NTRK1	**1q23.1**	●					✔	✔	CPS-ab	Pathways in cancer	◊	9

CADM3	**1q23.2**	●						✔	CMP-ab	C1-set| C2-set_2| I-sset| V-set| ig	○○○○○	21
CACNA1E	1q25.3	●●			✔			✔	CPS-s	Type II diabetes mellitus	◊	1
PLXNA2	1q32.2	●●	✔	✔	✔	✔	✔	✔	CPS-ab	Axon guidance	◊◊◊	2
CAPN2	1q41	●●			✔		✔	✔	CMP-ab	Calpain_III| Peptidase_C2	○○○○○	38
CAPN8	1q41	●●		✔	✔		✔	✔	CMP-ab	Calpain_III| Peptidase_C2	○○○○○	38
AIDA	1q41	●			✔	✔	✔	✔	MIR-ab	MI0000270	◊◊	1
PYCR2	1q42.12	●					✔	✔	CPS-s	Metabolic pathways	◊◊◊◊	1
EPHX1	1q42.12	●		✔	✔	✔	✔	✔	CPS-ab	Eicosanoid Metabolism	◊	5
H3F3A	1q42.12	●						✔	CPS-ab	Systemic lupus erythematosus	◊◊◊	3
CAPN9	1q42.2	●●			✔		✔	✔	CMP-ab	Calpain_III| Peptidase_C2	○○○○○	38
EGLN1	1q42.2	●●					✔	✔	CPS-ab	Pathways in cancer	◊	9
NID1	1q42.3	●					✔	✔	CMP-s	4040 Ldl_recept_b	●●	3
ERO1LB	1q42.3	●				✔	✔	✔	PPI-ab	8602, 5685, 55226, 10885	◊◊	316
CHRM3	1q43	●						✔	CMP-ab	7TM_GPCR_Srsx| 7TM_GPCR_Srx| 7tm_1	○○○○○	65
ACTN2	1q43	●						✔	PPI-s	4846	◊◊	17
HNRNPU	1q44	●			✔			✔	PPI-s	3667	◊◊◊◊	7
ADAM17	2p25.1	●						✔	CMP-ab	Disintegrin| Pep_M12B_propep| Reprolysin	○○○○○	52
LPIN1	2p25.1	●						✔	CMP-ab	LNS2| Lipin_N	○○○○○	68
MBOAT2	2p25.1	●					✔	✔	CPS-s	Metabolic pathways	◊◊◊◊	1
ASAP2	2p25.1	●			✔	✔	✔	✔	CPS-ab	ADP-sRibosylation Factor	◊◊◊	3
ID2	2p25.1	●			✔			✔	CPS-ab	TGF-beta signaling pathway	◊	16
PNPT1	2p16.1	●						✔	CMP-ab	RNase_PH| RNase_PH_C	○○○○○	74
CTNNA2	2p12	●			✔				CPS-ab	Pathways in cancer	◊◊◊	2
NPAS2	2q11.2	●						✔	CMP-ab	HLH| PAS| PAS_3	○○○○○	51
EDAR	2q13	●						✔	CPS-s	Cytokine-cytokine receptor interaction	◊◊◊	2
GLI2	2q14.2	●●●			✔		✔	✔	CPS-ab	Pathways in cancer	◊◊◊	2
ARL5A	2q23.3	●					✔	✔	CMP-ab	Arf| Gtr1_RagA| Miro| Ras| SRPRB	○○○○○	5
CACNB4	2q23.3	●	✔	✔	✔	✔	✔	✔	CPS-ab	Arrhythmogenic right ventricular cardiomyopathy (ARVC)	◊	4
STAM2	2q23.3	●					✔	✔	CPS-ab	Jak-STAT signaling pathway	◊	10
UPP2	2q24.1	●●						✔	CPS-s	Metabolic pathways	◊◊◊◊	1
ABCA12	2q35	●	✔	✔	✔	✔	✔	✔	CMP-s	19 ABC_tran	●	2
ATIC	2q35	●			✔			✔	CPS-s	Metabolic pathways	◊◊◊◊	1
BARD1	2q35	●					✔	✔	CPS-ab	BRCA1-dependent Ub-ligase activity	◊◊	6
FN1	2q35	●						✔	PPI-s	4018	◊◊◊◊	11
IRS1	**2q36.3**	●●		✔	✔			✔	CPS-s	**2q36** Insulin signaling pathway	◊	1
HTR2B	2q37.1	●						✔	CMP-ab	7TM_GPCR_Srsx| 7TM_GPCR_Srv| 7tm_1	○○○○○	69
RAB17	2q37.3	●			✔		✔	✔	CMP-ab	Arf| Gtr1_RagA| Miro| Ras	○○○○○	11
KLHL30	2q37.3	●						✔	CMP-ab	BACK| BTB| Kelch_1	○○○○○	50
CAPN10	2q37.3	●				✔	✔	✔	CMP-ab	Calpain_III| Peptidase_C2	○○○○○	38
SNED1	2q37.3	●						✔	CMP-ab	EGF| EGF_2| EGF_CA	○○○○○	58
RNPEPL1	2q37.3	●	✔	✔	✔	✔	✔	✔	CMP-ab	Peptidase_M1	○○○○	17
SCLY	2q37.3	●						✔	CPS-s	Metabolic pathways	◊◊◊◊	1
AGXT	2q37.3	●						✔	CPS-s	Metabolic pathways	◊◊◊◊	1
GPC1	2q37.3	●					✔	✔	PPI-ab	7422, 2885, 80781	◊	53
SRGAP3	**3p25.3**	●						✔	CMP-ab	**3pter-p21, 3p21.2-p14.1** FCH| RhoGAP| SH3_1| SH3_2	○○○○○	32
VHL	**3p25.3**	●						✔	CPS-s	Hypoxia-Inducible Factor in the Cardiovascular System	◊◊	3
ARPC4	**3p25.3**	●			✔	✔	✔	✔	CPS-ab	Bacterial invasion of epithelial cells	◊	19
FANCD2	**3p25.3**	●					✔	✔	CPS-ab	BRCA1-dependent Ub-ligase activity	◊◊	6
CAMK1	**3p25.3**	●			✔	✔	✔	✔	CPS-ab	fMLP induced chemokine gene expression in HMC-1 cells	◊	15
IRAK2	**3p25.3**	●						✔	CPS-ab	Tuberculosis	◊◊◊	5
OXSM	**3p24.2**	●						✔	CPS-s	Metabolic pathways	◊◊◊◊	1
CX3CR1	**3p22.2**	●●			✔		✔	✔	CPS-s	Cytokine-cytokine receptor interaction	◊◊◊	2
MLH1	**3p22.2**	●					✔	✔	CPS-ab	Pathways in cancer	◊	9
CCR8	**3p22.1**	●●					✔	✔	CMP-s	1524 7TM_GPCR_Srsx| 7tm_1	●	6
RPSA	**3p22.1**	●●		✔	✔	✔	✔	✔	PPI-ab	11128, 3778	◊	43
CCR3	**3p21.31**	●			✔		✔	✔	CMP-s	1524 7TM_GPCR_Srsx| 7tm_1	●	7
CCR1	**3p21.31**	●					✔	✔	CMP-s	1524 7TM_GPCR_Srsx| 7tm_1	●	9
EXOSC7	**3p21.31**	●					✔	✔	CMP-ab	RNase_PH| RNase_PH_C	○○○○○	74
CCR9	**3p21.31**	●					✔	✔	CPS-s	Cytokine-cytokine receptor interaction	◊◊◊	2
CCR2	**3p21.31**	●						✔	CPS-s	Cytokine-cytokine receptor interaction	◊◊◊	2
CCR5	**3p21.31**	●						✔	CPS-s	Cytokine-cytokine receptor interaction	◊◊◊	2
XCR1	**3p21.31**	●			✔		✔	✔	CPS-s	Cytokine-cytokine receptor interaction	◊◊◊	2
CXCR6	**3p21.31**	●					✔	✔	CPS-s	Cytokine-cytokine receptor interaction	◊◊◊	2

LARS2	**3p21.31**	●			✔	✔	✔	✔	CPS-ab	Valine, leucine and isoleucine biosynthesis	◊	3
SACM1L	**3p21.31**	●					✔	✔	PPI-ab	10564	◊◊◊	98
FYCO1	**3p21.31**	●					✔	✔	PPI-ab	26227, 3861, 506, 2023, 3320, 3326, 3868, 3860, 1832, 6950, 3187, 4174, 5984, 3857, 47, 4637, 6155, 6632, 6229, 6160, 5478, 2969, 3192, 6128, 708, 5708, 5790, 9859, 54800, 25902, 22879, 10808, 81631, 51493	◊	31
IL17RB	**3p21.1**	●						✔	CPS-s	Cytokine-cytokine receptor interaction	◊◊◊	2
CACNA1D	**3p21.1**	●						✔	CPS-s	Type II diabetes mellitus	◊◊	4
CACNA2D3	**3p14.3**	●	✔	✔	✔	✔	✔	✔	CPS-ab	Arrhythmogenic right ventricular cardiomyopathy (ARVC)	◊	4
FHIT	**3p14.2**	●	✔	✔	✔	✔	✔	✔	CPS-ab	Small cell lung cancer	◊◊◊	4
GBE1	3p12.2	●	✔	✔	✔	✔	✔	✔	CPS-s	Metabolic pathways	◊◊◊◊	1
CADM2	3p12.1	●			✔		✔	✔	CMP-ab	C2-set_2| I-sset| V-set| ig	○○○○○	17
B4GALT4	3q13.32	●						✔	CPS-s	Metabolic pathways	◊◊◊◊	1
CD86	3q13.33	●						✔	CPS-ab	Systemic lupus erythematosus	◊◊◊	3
CASR	3q21.1	●						✔	CMP-ab	7tm_3| ANF_receptor| NCD3G	○○○○○	45
MYLK	3q21.1	●●●						✔	CMP-ab	I-sset| Pkinase| Pkinase_Tyr| V-set| ig	○○○○○	34
KPNA1	3q21.1	●					✔	✔	PPI-ab	5896, 7398, 672, 1434, 5897, 8543, 862	◊◊	47
ITGB5	3q21.2	●●●						✔	CMP-ab	EGF_2| Integrin_B_tail| Integrin_b_cyt| Integrin_beta	○○○○○	7
KALRN	3q21.2	●●●	✔	✔	✔	✔	✔	✔	CMP-ab	I-sset| V-set| ig	○○○○○	6
UMPS	3q21.2	●●●						✔	CPS-s	Metabolic pathways	◊◊◊◊	1
RASA2	3q23	●						✔	CMP-ab	C2| PH| RasGAP	○○○○○	70
AGTR1	3q24	●●			✔				CPS-ab	Angiotensin-converting enzyme 2 regulates heart function	◊◊	6
IGSF10	3q25.1	●●						✔	CMP-ab	I-sset| V-set| ig	○○○○○	67
MECOM	3q26.2	●	✔	✔	✔	✔	✔	✔	CPS-ab	Pathways in cancer	◊	5
PLD1	3q26.31	●	✔	✔	✔	✔	✔	✔	CPS-s	Metabolic pathways	◊◊◊◊	1
KLHL6	3q27.1	●			✔		✔	✔	CMP-ab	BACK| BTB| Kelch_1	○○○○○	22
KLHL24	3q27.1	●					✔	✔	CMP-ab	BACK| BTB| Kelch_1| Kelch_2	○○○○○	10
EIF4G1	3q27.1	●						✔	CMP-ab	MA3| MIF4G| W2	○○○○○	33
POLR2H	3q27.1	●						✔	CPS-s	Metabolic pathways	◊◊◊◊	1
ALG3	3q27.1	●						✔	CPS-s	Metabolic pathways	◊◊◊◊	1
EIF2B5	3q27.1	●					✔	✔	CPS-s	VEGF, Hypoxia, and Angiogenesis	◊	4
DVL3	3q27.1	●					✔	✔	CPS-ab	Pathways in cancer	◊	9
ST6GAL1	3q27.3	●	✔	✔	✔	✔	✔	✔	CPS-s	Metabolic pathways	◊◊◊◊	1
ADIPOQ	3q27.3	●					✔	✔	CPS-s	Type II diabetes mellitus	◊◊	4
ADRA2C	4p16.2	●					✔	✔	CMP-ab	7TM_GPCR_Srsx| 7TM_GPCR_Srx| 7tm_1	○○○○○	32
HGFAC	4p16.2	●						✔	CMP-ab	EGF| Kringle| Trypsin| fn1	○○○○○	18
PCDH7	4p15.1	●		✔	✔			✔	CMP-ab	Cadherin| Cadherin_2| Protocadherin	○○○○○	20
PGM2	4p14	●					✔	✔	CPS-s	Metabolic pathways	◊◊◊◊	1
LIMCH1	4p13	●	✔	✔	✔	✔	✔	✔	CMP-ab	CH	○○○○	18
GNPDA2	4p13	●●						✔	CPS-s	Metabolic pathways	◊◊◊◊	1
UCHL1	4p13	●					✔	✔	PPI-ab	3320, 1027, 10048, 23043, 7189, 3872	◊◊	62
KIT	4q12	●		✔	✔		✔	✔	CPS-s	Cytokine-cytokine receptor interaction	◊◊◊	2
PDGFRA	4q12	●			✔			✔	CPS-s	Cytokine-cytokine receptor interaction	◊◊◊	2
IL21	4q27	●					✔	✔	CPS-s	Cytokine-cytokine receptor interaction	◊◊◊	2
IL2	4q27	●						✔	CPS-s	Cytokine-cytokine receptor interaction	◊◊◊	2
FGF2	4q28.1	●			✔		✔	✔	CPS-ab	Pathways in cancer	◊◊◊	2
SPATA5	4q28.1	●					✔	✔	PPI-ab	11137, 23560	◊	347
PDGFC	4q32.1	●			✔			✔	CPS-s	Cytokine-cytokine receptor interaction	◊◊◊	2
MFAP3L	4q33	●●●●					✔	✔	CMP-ab	I-sset| V-set| ig	○○○○○	34
AADAT	4q33	●●●●					✔	✔	CPS-s	Metabolic pathways	◊◊◊◊	1
ACSL1	4q35.1	●						✔	CPS-s	Metabolic pathways	◊◊◊◊	1
LPCAT1	5p15.33	●					✔	✔	CPS-s	Metabolic pathways	◊◊◊◊	1
NDUFS6	5p15.33	●			✔		✔	✔	CPS-s	Metabolic pathways	◊◊◊◊	1
SKP2	5p13.2	●●			✔			✔	CPS-ab	Pathways in cancer	◊◊◊	2
FGF10	5p12	●●●●						✔	CPS-ab	Melanoma	◊	5
ARL15	5q11.2	●	✔	✔	✔	✔	✔	✔	CMP-ab	Arf| Gtr1_RagA| Miro| Ras| SRPRB	○○○○○	4
NDUFS4	5q11.2	●						✔	CPS-s	Metabolic pathways	◊◊◊◊	1
HTR1A	5q12.2	●			✔				CMP-ab	7TM_GPCR_Srsx| 7TM_GPCR_Srx| 7tm_1	○○○○○	25
IQGAP2	5q13.3	●	✔	✔	✔	✔	✔	✔	CMP-ab	CH	○○○○	18
RASA1	5q14.3	●			✔			✔	CPS-s	Insulin Signaling Pathway	◊	2
COX7C	5q14.3	●						✔	CPS-s	Metabolic pathways	◊◊◊◊	1
ERAP2	5q15	●	✔	✔	✔	✔	✔	✔	CMP-ab	Peptidase_M1	○○○○	17
ERAP1	5q15	●			✔		✔	✔	PPI-ab	64167, 10564	◊	36
ST8SIA4	5q21.1	●●●			✔		✔	✔	CPS-ab	Steps in the Glycosylation of Mammalian	◊◊◊	3

									N-linked Oligosaccarides		
MAN2A1	5q21.3	●●	✓	✓	✓	✓	✓	✓	CPS-s	Metabolic pathways	◊◊◊◊	1
CXCL14	5q31.1	●						✓	CPS-s	Cytokine-cytokine receptor interaction	◊◊◊	2
IL9	5q31.1	●					✓	✓	CPS-s	Cytokine-cytokine receptor interaction	◊◊◊	2
TGFBI	5q31.2	●		✓	✓	✓	✓	✓	CMP-ab	Fasciclin	oooo	30
SMAD5	5q31.2	●			✓		✓	✓	CMP-ab	IRF-3| MH1| MH2	ooooo	10
C5orf4	5q33.2	●						✓	CPS-s	Metabolic pathways	◊◊◊◊	1
GRM6	5q35.3	●					✓	✓	CMP-ab	7tm_3| ANF_receptor| NCD3G	ooooo	45
ADAMTS2	5q35.3	●	✓	✓	✓	✓	✓	✓	CMP-ab	ADAM_spacer1| Pep_M12B_propep| Reprolysin| TSP_1	ooooo	1
CANX	5q35.3	●						✓	PPI-s	4018	◊◊◊◊	12
BMP6	6p24.3	●		✓	✓	✓	✓	✓	CPS-s	Cytokine-cytokine receptor interaction	◊◊◊	2
EDN1	6p24.1	●						✓	CPS-s	Hypoxia-Inducible Factor in the Cardiovascular System	◊◊	3
MDGA1	6p21.2	●	✓	✓	✓	✓	✓	✓	CMP-ab	I-sset| V-set| ig	ooooo	6
PIM1	6p21.2	●						✓	CPS-ab	Jak-STAT signaling pathway	◊	10
CAPN11	6p21.1	●●					✓	✓	CMP-ab	Calpain_III| Peptidase_C2	ooooo	38
AARS2	6p21.1	●●						✓	CMP-ab	tRNA-synt_2c| tRNA_SAD	ooooo	71
VEGFA	6p21.1	●●					✓	✓	CPS-s	Hypoxia-Inducible Factor in the Cardiovascular System	◊◊	2
POLR1C	6p21.1	●●						✓	CPS-s	Metabolic pathways	◊◊◊◊	1
HSP90AB1	6p21.1	●●						✓	PPI-s	3667	◊◊◊◊	2
XPO5	6p21.1	●●						✓	PPI-ab	4609, 8793	◊◊	406
SUPT3H	6p12.3	●			✓		✓	✓	PPI-ab	1105, 4609, 10474, 6883	◊	67
EYS	6q12	●	✓	✓	✓	✓	✓	✓	CMP-ab	EGF| Laminin_G_1| Laminin_G_2	ooooo	3
FUT9	6q16.1	●●●●	✓	✓	✓	✓	✓	✓	CPS-s	Metabolic pathways	◊◊◊◊	1
GRIK2	6q16.3	●			✓				CMP-ab	ANF_receptor| Lig_chan| Lig_chan-Glu_bd| SBP_bac_3	ooooo	3
WASF1	6q21	●			✓		✓	✓	CPS-ab	Rac 1 cell motility signaling pathway	◊◊◊	5
RSPO3	6q22.33	●		✓	✓	✓	✓	✓	CMP-ab	TSP_1	oooo	31
SGK1	6q23.2	●				✓	✓	✓	CMP-ab	Pkinase| Pkinase_C| Pkinase_Tyr	ooooo	77
LRP11	6q25.1	●	✓	✓	✓	✓	✓	✓	CMP-s	4040 Ldl_recept_a	●●	1
KATNA1	6q25.1	●					✓	✓	CMP-ab	AAA| AAA_2| RuvB_N| Vps4_C	ooooo	11
<u>*MTHFD1L*</u>	6q25.1	●●●	✓	✓	✓	✓	✓	✓	CPS-s	Metabolic pathways	◊◊◊◊	1
LATS1	6q25.1	●					✓	✓	PPI-ab	4609, 3984, 5879	◊	106
SYNE1	6q25.2	●	✓	✓	✓	✓	✓	✓	CMP-ab	CH	oooo	18
ACAT2	6q25.3	●					✓	✓	CPS-s	Metabolic pathways	◊◊◊◊	1
PLG	6q26	●						✓	CMP-s	4018 DUF1986| Kringle| Trypsin	●●●●	1
LPA	6q26	●					✓	✓	CMP-ab	DUF1986| Kringle| Trypsin	ooooo	53
AGPAT4	6q26	●						✓	CPS-s	Metabolic pathways	◊◊◊◊	1
RAC1	7p22.1	●			✓		✓	✓	CPS-s	Phagosome	◊	3
CYTH3	7p22.1	●			✓	✓	✓	✓	CPS-ab	ADP-sRibosylation Factor	◊◊◊	3
EIF2AK1	7p22.1	●						✓	CPS-ab	Regulation of eIF2	◊	14
DGKB	7p21.2	●	✓	✓	✓	✓	✓	✓	CPS-s	Metabolic pathways	◊◊◊◊	1
RALA	7p14.1	●						✓	CMP-ab	Arf| GTP_EFTU| Miro| Ras	ooooo	30
DDX56	7p13	●	✓	✓	✓	✓	✓	✓	CMP-ab	DEAD| Helicase_C| ResIII	ooooo	21
TBRG4	7p13	●						✓	CMP-ab	FAST_1| FAST_2| RAP	ooooo	23
OGDH	7p13	●	✓	✓	✓	✓	✓	✓	CPS-s	Metabolic pathways	◊◊◊◊	1
PGAM2	7p13	●						✓	CPS-s	Metabolic pathways	◊◊◊◊	1
POLD2	7p13	●						✓	CPS-s	Metabolic pathways	◊◊◊◊	1
GCK	7p13	●						✓	CPS-s	Metabolic pathways	◊◊◊◊	1
CAMK2B	7p13	●					✓	✓	CPS-ab	Olfactory transduction	◊◊◊◊	1
H2AFV	7p13	●			✓		✓	✓	CPS-ab	Systemic lupus erythematosus	◊◊	3
										**7q11.2**		
LIMK1	**7q11.23**	●●●					✓	✓	CPS-ab	Rac 1 cell motility signaling pathway	◊◊	5
LAT2	**7q11.23**	●●●				✓	✓	✓	PPI-ab	5336, 2885	◊◊	6
CD36	**7q21.11**	●●			✓		✓	✓	CPS-s	Phagosome	◊	3
SEMA3A	**7q21.11**	●		✓	✓	✓	✓	✓	CPS-ab	Axon guidance	◊◊◊	1
SEMA3C	**7q21.11**	●●	✓	✓	✓	✓	✓	✓	CPS-ab	Axon guidance	◊◊◊	2
										**7q21.3**		
PON1	**7q21.3**	-										
PON2	**7q21.3**	-										
RELN	7q22.1	●	✓	✓	✓	✓	✓	✓	CPS-s	ECM-receptor interaction	◊	1
DOCK4	7q31.1	●	✓	✓	✓	✓	✓	✓	CMP-ab	Ded_cyto	oooo	16
BPGM	7q33	●		✓	✓	✓	✓	✓	CPS-s	Metabolic pathways	◊◊◊◊	1
AKR1B10	7q33	●					✓	✓	CPS-s	Metabolic pathways	◊◊◊◊	1
AKR1B1	7q33	●			✓		✓	✓	CPS-s	Metabolic pathways	◊◊◊◊	1
										**7q36**		
ABP1	**7q36.1**	●			✓	✓	✓	✓	CMP-ab	Cu_amine_oxid| Cu_amine_oxidN2| Cu_amine_oxidN3	ooooo	19
FASTK	**7q36.1**	●						✓	CMP-ab	FAST_1| FAST_2| RAP	ooooo	23

NOS3	**7q36.1**	●					✔	✔	CPS-s	Metabolic pathways	◊◊◊◊	1
ATP6V0E2	**7q36.1**	●						✔	CPS-s	Metabolic pathways	◊◊◊◊	1
CHPF2	**7q36.1**	●						✔	CPS-s	Metabolic pathways	◊◊◊◊	1
ABCF2	**7q36.1**	●						✔	PPI-ab	6157, 84940	◊◊◊	234
SHH	**7q36.3**	●			✔			✔	CPS-ab	Pathways in cancer	◊◊◊	2
ASAH1	8p22	●					✔	✔	CPS-s	Metabolic pathways	◊◊◊◊	1
NAT1	8p22	●			✔		✔	✔	CPS-s	Metabolic pathways	◊◊◊◊	1
NAT2	8p22	●					✔	✔	CPS-s	Metabolic pathways	◊◊◊◊	1
TUSC3	8p22	●●			✔				CPS-ab	N-Glycan biosynthesis	◊	12
										**8p21-p12**		
TNFRSF10D	**8p21.3**	●						✔	CPS-s	Cytokine-cytokine receptor interaction	◊◊◊	2
TNFRSF10A	**8p21.3**	●					✔	✔	CPS-s	Cytokine-cytokine receptor interaction	◊◊◊	2
TNFRSF10C	**8p21.3**	●						✔	CPS-s	Cytokine-cytokine receptor interaction	◊◊◊	2
TNFRSF10B	**8p21.3**	●						✔	CPS-s	Cytokine-cytokine receptor interaction	◊◊◊	2
ZNF703	**8p12**	●			✔			✔	CMP-ab	nlz1	○○○○	78
ANK1	8p11.21	●						✔	CMP-ab	Ank| Death| ZU5	○○○○○	60
IKBKB	8p11.21	●			✔		✔	✔	CPS-s	Type II diabetes mellitus	◊	1
PLAT	8p11.21	●		✔	✔	✔	✔	✔	CPS-ab	Platelet Amyloid Precursor Protein Pathway	◊◊	2
JPH1	8q21.11	●	✔	✔	✔	✔	✔	✔	PPI-ab	4088	◊	5
ATP6V0D2	8q21.3	●	✔	✔	✔	✔	✔	✔	CPS-s	Metabolic pathways	◊◊◊◊	1
RUNX1T1	8q21.3	●					✔	✔	CPS-ab	Pathways in cancer	◊	9
SQLE	8q24.13	●						✔	CPS-s	Metabolic pathways	◊◊◊◊	1
MYC	8q24.21	●			✔		✔	✔	CPS-ab	Pathways in cancer	◊◊◊	2
ST3GAL1	8q24.22	●			✔		✔	✔	CPS-s	Metabolic pathways	◊◊◊◊	1
BAI1	8q24.3	●●		✔	✔		✔	✔	CMP-ab	7tm_2| DUF3497| GPS	○○○○○	4
JRK	8q24.3	●					✔	✔	CMP-ab	CENP-sB_N| DDE| Transposase_Tc5	○○○○○	29
TIGD5	8q24.3	●						✔	CMP-ab	CENP-sB_N| DDE| Transposase_Tc5	○○○○○	29
CYP11B2	8q24.3	●						✔	CPS-s	Metabolic pathways	◊◊◊◊	1
CYP11B1	8q24.3	●						✔	CPS-s	Metabolic pathways	◊◊◊◊	1
PYCRL	8q24.3	●						✔	CPS-s	Metabolic pathways	◊◊◊◊	1
NAPRT1	8q24.3	●						✔	CPS-s	Metabolic pathways	◊◊◊◊	1
TSTA3	8q24.3	●						✔	CPS-s	Metabolic pathways	◊◊◊◊	1
MAFA	8q24.3	●					✔	✔	CPS-s	Type II diabetes mellitus	◊◊	4
ARC	8q24.3	●●			✔		✔	✔	PPI-ab	3866, 2904	◊	210
DOCK8	9p24.3	●	✔	✔	✔	✔	✔	✔	CMP-ab	Ded_cyto	○○○○	16
CD274	9p24.1	●						✔	CMP-ab	C1-set| C2-set_2| V-set| ig	○○○○○	41
JAK2	9p24.1	●						✔	PPI-s	3667	◊◊◊◊	8
MTAP	9p21.3	●●●●					✔	✔	CPS-s	Metabolic pathways	◊◊◊◊	1
CDKN2A	9p21.3	●●●●				✔	✔	✔	CPS-ab	Non-small cell lung cancer	◊	6
CDKN2B	9p21.3	●●●●			✔	✔	✔	✔	CPS-ab	Pathways in cancer	◊◊◊	2
										**9q22-q31**		
CORO2A	**9q22.33**	●●	✔	✔	✔	✔	✔	✔	CMP-ab	DUF1899| DUF1900| WD40	○○○○○	6
NANS	**9q22.33**	●●			✔		✔	✔	CPS-s	Metabolic pathways	◊◊◊◊	1
TRIM14	**9q22.33**	●●				✔	✔	✔	PPI-ab	2885	◊	14
NCBP1	**9q22.33**	●●						✔	PPI-ab	6780, 3187, 6632, 5395, 1982, 1981, 8175, 23008, 84078, 26512	◊◊	158
ABCA1	**9q31.2**	-										
EPB41L4B	**9q31.3**	●						✔	CMP-ab	FA| FERM_C| FERM_M| FERM_N	○○○○○	15
PTPN3	**9q31.3**	●						✔	CMP-ab	FERM_C| FERM_M| FERM_N	○○○○○	40
UGCG	**9q31.3**	●		✔	✔		✔	✔	CPS-s	Metabolic pathways	◊◊◊◊	1
IKBKAP	**9q31.3**	●	✔	✔	✔	✔	✔	✔	CPS-ab	CD40L Signaling Pathway	◊◊	7
PTGS1	9q33.2	●●						✔	CPS-s	Metabolic pathways	◊◊◊◊	1
OR1B1	9q33.2	●●			✔	✔	✔	✔	CPS-ab	Olfactory transduction	◊◊◊	1
OR1L3	9q33.2	●●	✔	✔	✔	✔	✔	✔	CPS-ab	Olfactory transduction	◊◊◊	1
OR1L4	9q33.2	●●				✔	✔	✔	CPS-ab	Olfactory transduction	◊	5
OR1L1	9q33.2	●●				✔	✔	✔	CPS-ab	Olfactory transduction	◊	5
OR1J1	9q33.2	●●					✔	✔	CPS-ab	Olfactory transduction	◊◊◊◊	1
OR1Q1	9q33.2	●●					✔	✔	CPS-ab	Olfactory transduction	◊◊◊◊	1
OR1K1	9q33.2	●●					✔	✔	CPS-ab	Olfactory transduction	◊◊◊◊	1
OR5C1	9q33.2	●●					✔	✔	CPS-ab	Olfactory transduction	◊◊◊◊	1
OR1L8	9q33.2	●●					✔	✔	CPS-ab	Olfactory transduction	◊◊◊◊	1
OR1J2	9q33.2	●●					✔	✔	CPS-ab	Olfactory transduction	◊◊◊◊	1
OR1N2	9q33.2	●●					✔	✔	CPS-ab	Olfactory transduction	◊◊◊◊	1
OR1N1	9q33.2	●●					✔	✔	CPS-ab	Olfactory transduction	◊◊◊◊	1
OR1J4	9q33.2	●●					✔	✔	CPS-ab	Olfactory transduction	◊◊◊◊	1
OR1L6	9q33.2	●●					✔	✔	CPS-ab	Olfactory transduction	◊◊◊◊	1
RABGAP1	9q33.2	●●						✔	PPI-ab	26227, 3861, 3690, 3689, 3320, 3326, 3868, 3860, 5870, 3187, 5245, 3857, 6155, 6632, 6229, 6160, 2969, 3192, 6128, 708, 6429, 3757, 11137, 25902,	◊	51

										6601, 51493, 3688		
ADAMTSL2	9q34.2	●						✔	CMP-ab	ADAM_spacer1| PLAC| TSP_1	○○○○○	38
ADARB2	10p15.3	●	✔	✔	✔	✔	✔	✔	CMP-ab	A_deamin| dsrm	○○○○○	4
IDI2	10p15.3	●					✔	✔	CPS-s	Metabolic pathways	◊◊◊◊	1
IDI1	10p15.3	●					✔	✔	CPS-s	Metabolic pathways	◊◊◊◊	1
FRMD4A	10p13	●	✔	✔	✔	✔	✔	✔	CMP-ab	FERM_C| FERM_M| FERM_N	○○○○○	2
NRP1	10p11.22	●		✔	✔	✔	✔	✔	CPS-ab	Axon guidance	◊◊◊	1
ITGB1	10p11.22	●						✔	PPI-s	948	◊◊◊◊	9
PRKG1	10q21.1	●	✔	✔	✔	✔	✔	✔	CPS-ab	Olfactory transduction	◊◊◊	1
ANK3	10q21.2	●	✔	✔	✔	✔	✔	✔	CMP-ab	Ank| Death| ZU5	○○○○○	60
CTNNA3	10q21.3	●	✔	✔	✔	✔	✔	✔	CPS-ab	Adherens junction	◊◊◊	1
ZNF503	10q22.2	●			✔				CMP-ab	nlz1	○○○○	78
POLR3A	10q22.3	●	✔	✔	✔	✔	✔	✔	CPS-s	Metabolic pathways	◊◊◊◊	1
TNKS2	10q23.32	●●						✔	CMP-ab	Ank| SAM_1| SAM_2	○○○○○	7
SORBS1	10q23.33	●●	✔	✔	✔	✔	✔	✔	CPS-s	Insulin signaling pathway	◊	1
ALDH18A1	10q23.33	●●					✔	✔	CPS-s	Metabolic pathways	◊◊◊◊	1
CYP2C9	10q23.33	●●						✔	CPS-s	Metabolic pathways	◊◊◊◊	1
CYP2C8	10q23.33	●●						✔	CPS-s	Metabolic pathways	◊◊◊◊	1
PNLIP	10q25.3	●						✔	CPS-s	Metabolic pathways	◊◊◊◊	1
PNLIPRP3	10q25.3	●						✔	CPS-s	Metabolic pathways	◊◊◊◊	1
TH	11p15.5	●						✔	CPS-s	Metabolic pathways	◊◊◊◊	1
INS	11p15.5	●						✔	CPS-s	Type II diabetes mellitus	◊◊	4
SBF2	11p15.4	●					✔	✔	CMP-ab	DENN| dDENN| uDENN	○○○○○	35
ST5	11p15.4	●						✔	CMP-ab	DENN| dDENN| uDENN	○○○○○	35
TRIM6	11p15.4	●					✔	✔	CMP-ab	SPRY| zf-B_box| zf-C3HC4	○○○○○	33
TRIM5	11p15.4	●	✔	✔	✔	✔	✔	✔	CMP-ab	SPRY| zf-B_box| zf-C3HC4	○○○○○	10
TRIM22	11p15.4	●			✔	✔	✔	✔	CMP-ab	SPRY| zf-B_box| zf-C3HC4	○○○○○	10
TRIM6-TRIM34	11p15.4	●				✔	✔	✔	CMP-ab	SPRY| zf-B_box| zf-C3HC4	○○○○○	10
TRIM34	11p15.4	●				✔	✔	✔	CMP-ab	SPRY| zf-B_box| zf-C3HC4	○○○○○	10
AMPD3	11p15.4	●		✔	✔	✔	✔	✔	CPS-s	Metabolic pathways	◊◊◊◊	1
OR10A3	11p15.4	●						✔	CPS-ab	Olfactory transduction	◊◊◊◊	2
OR10A6	11p15.4	●						✔	CPS-ab	Olfactory transduction	◊◊◊◊	2
OR56A5	11p15.4	●						✔	CPS-ab	Olfactory transduction	◊◊◊◊	2
OR56A1	11p15.4	●						✔	CPS-ab	Olfactory transduction	◊◊◊◊	2
OR56A3	11p15.4	●						✔	CPS-ab	Olfactory transduction	◊◊◊◊	2
OR5P3	11p15.4	●						✔	CPS-ab	Olfactory transduction	◊◊◊◊	2
OR51V1	11p15.4	●						✔	CPS-ab	Olfactory transduction	◊◊◊◊	2
OR51M1	11p15.4	●						✔	CPS-ab	Olfactory transduction	◊◊◊◊	2
OR56A4	11p15.4	●						✔	CPS-ab	Olfactory transduction	◊◊◊◊	2
OR51B4	11p15.4	●						✔	CPS-ab	Olfactory transduction	◊◊◊◊	2
OR56B4	11p15.4	●						✔	CPS-ab	Olfactory transduction	◊◊◊◊	2
OR51B2	11p15.4	●						✔	CPS-ab	Olfactory transduction	◊◊◊◊	2
OR51B6	11p15.4	●						✔	CPS-ab	Olfactory transduction	◊◊◊◊	2
OR5P2	11p15.4	●						✔	CPS-ab	Olfactory transduction	◊◊◊◊	2
OR52L1	11p15.4	●						✔	CPS-ab	Olfactory transduction	◊◊◊◊	2
OR51Q1	11p15.4	●						✔	CPS-ab	Olfactory transduction	◊◊◊◊	2
OR52B2	11p15.4	●						✔	CPS-ab	Olfactory transduction	◊◊◊◊	2
OR51B5	11p15.4	●						✔	CPS-ab	Olfactory transduction	◊◊◊◊	2
OR52E8	11p15.4	●					✔	✔	CPS-ab	Olfactory transduction	◊◊◊◊	1
OR52E6	11p15.4	●					✔	✔	CPS-ab	Olfactory transduction	◊◊◊◊	1
OR52B6	11p15.4	●					✔	✔	CPS-ab	Olfactory transduction	◊◊◊◊	1
OR52N1	11p15.4	●					✔	✔	CPS-ab	Olfactory transduction	◊◊◊◊	1
OR56B1	11p15.4	●					✔	✔	CPS-ab	Olfactory transduction	◊◊◊◊	1
OR52N2	11p15.4	●					✔	✔	CPS-ab	Olfactory transduction	◊◊◊◊	1
OR52E4	11p15.4	●					✔	✔	CPS-ab	Olfactory transduction	◊◊◊◊	1
OR51I1	11p15.4	●					✔	✔	CPS-ab	Olfactory transduction	◊◊◊◊	1
OR51I2	11p15.4	●					✔	✔	CPS-ab	Olfactory transduction	◊◊◊◊	1
OR52D1	11p15.4	●					✔	✔	CPS-ab	Olfactory transduction	◊◊◊◊	1
OR52N5	11p15.4	●					✔	✔	CPS-ab	Olfactory transduction	◊◊◊◊	1
OR52H1	11p15.4	●					✔	✔	CPS-ab	Olfactory transduction	◊◊◊◊	1
OR52N4	11p15.4	●					✔	✔	CPS-ab	Olfactory transduction	◊◊◊◊	1
KCNQ1	11p15.4	●	✔	✔	✔	✔	✔	✔	CPS-ab	Vibrio cholerae infection	◊◊	3
PARVA	11p15.3	●●●●	✔	✔	✔	✔	✔	✔	CMP-ab	CH	○○○○	18
MICAL2	11p15.3	●●●●	✔	✔	✔	✔	✔	✔	CMP-ab	CH	○○○○	18
EIF4G2	11p15.3	●						✔	CMP-ab	MA3| MIF4G| W2	○○○○○	33
CTR9	11p15.3	●						✔	PPI-ab	80349	◊◊◊◊	204
MYOD1	11p15.1	●					✔	✔	CMP-ab	Basic| HLH| Myf5	○○○○○	11
OTOG	11p15.1	●			✔	✔	✔	✔	CMP-ab	C8| TIL| VWD	○○○○○	9
KCNC1	11p15.1	●					✔	✔	CMP-ab	Ion_trans| Ion_trans_2| K_tetra	○○○○○	56
PIK3C2A	11p15.1	●					✔	✔	CPS-s	Metabolic pathways	◊◊◊◊	1

KCNJ11	11p15.1	●					✓	✓	CPS-s	Type II diabetes mellitus	◊◊	4
ABCC8	11p15.1	●				✓	✓	✓	CPS-s	Type II diabetes mellitus	◊◊	4
USH1C	11p15.1	●	✓	✓	✓	✓	✓	✓	CPS-ab	Chaperones modulate interferon Signaling Pathway	◊	11
LDLRAD3	11p13	●						✓	CMP-s	4040 Ldl_recept_a	●●	6
CAT	11p13	●			✓		✓	✓	CPS-s	Metabolic pathways	◊◊◊◊	1
APIP	11p13	●			✓			✓	CPS-s	Metabolic pathways	◊◊◊◊	1
PDHX	11p13	●						✓	CPS-s	Metabolic pathways	◊◊◊◊	1
TRAF6	11p12	●					✓	✓	CPS-ab	Toxoplasmosis	◊◊	4
EXT2	11p11.2	●	✓	✓	✓	✓	✓	✓	CPS-s	Metabolic pathways	◊◊◊◊	1
HSD17B12	11p11.2	●						✓	CPS-s	Metabolic pathways	◊◊◊◊	1
CD82	11p11.2	●						✓	PPI-ab	3689, 3688, 4179, 2065, 3675, 4927, 23118, 821	◊◊	107
MS4A2	11q12.1	●			✓		✓	✓	PPI-ab	2885, 3320	◊	52
CORO1B	11q13.1	●						✓	CMP-ab	DUF1899| DUF1900| WD40	ooooo	43
RPS6KB2	11q13.1	●						✓	CMP-ab	Pkinase| Pkinase_C| Pkinase_Tyr	ooooo	77
RPS6KA4	11q13.1	●						✓	CMP-ab	Pkinase| Pkinase_C| Pkinase_Tyr	ooooo	77
CLCF1	11q13.1	●						✓	CPS-s	Cytokine-cytokine receptor interaction	◊◊◊	2
VEGFB	11q13.1	●					✓	✓	CPS-s	Cytokine-cytokine receptor interaction	◊◊◊	2
COX8A	11q13.1	●				✓	✓	✓	CPS-s	Metabolic pathways	◊◊◊◊	1
POLD4	11q13.1	●						✓	CPS-s	Metabolic pathways	◊◊◊◊	1
PLCB3	11q13.1	●						✓	CPS-s	Metabolic pathways	◊◊◊◊	1
AIP	11q13.1	●					✓	✓	CPS-ab	Ahr Signal Transduction Pathway	◊	10
TRMT112	11q13.1	●						✓	PPI-ab	10474, 2035, 7428, 7327, 23043, 7189	◊	190
MACROD1	11q13.1	●			✓	✓	✓	✓	PPI-ab	2035	◊	10
ALDH3B2	11q13.2	●				✓	✓	✓	CPS-s	Metabolic pathways	◊◊◊◊	1
NDUFV1	11q13.2	●			✓		✓	✓	CPS-s	Metabolic pathways	◊◊◊◊	1
CHKA	11q13.2	●						✓	CPS-s	Metabolic pathways	◊◊◊◊	1
TCIRG1	11q13.2	●						✓	CPS-s	Metabolic pathways	◊◊◊◊	1
ALDH3B1	11q13.2	●						✓	CPS-s	Metabolic pathways	◊◊◊◊	1
NDUFS8	11q13.2	●						✓	CPS-s	Metabolic pathways	◊◊◊◊	1
GSTP1	11q13.2	●					✓	✓	CPS-ab	Pathways in cancer	◊	9
SHANK2	11q13.3	●					✓	✓	CMP-ab	SAM_1| SAM_2| SH3_2	ooooo	37
RAB6A	11q13.4	●●			✓	✓	✓	✓	CMP-ab	Arf| Gtr1_RagA| Miro| Ras	ooooo	11
RELT	11q13.4	●●					✓	✓	CPS-s	Cytokine-cytokine receptor interaction	◊◊◊	2
NADSYN1	11q13.4	●			✓		✓	✓	CPS-s	Metabolic pathways	◊◊◊◊	1
DHCR7	11q13.4	●					✓	✓	CPS-s	Metabolic pathways	◊◊◊◊	1
GRIA4	11q22.3	●						✓	CMP-ab	ANF_receptor| Lig_chan| Lig_chan-Glu_bd| SBP_bac_3	ooooo	13
KBTBD3	11q22.3	●			✓		✓	✓	CMP-ab	BACK| BTB| Kelch_1	ooooo	22
RDX	11q22.3	●			✓		✓	✓	CMP-ab	FERM_C| FERM_M| FERM_N	ooooo	14
										**11q23**		
MMP3	**11q22.3**	-										
APOA1	**11q23.3**	●						✓	PPI-s	19, 5444	◊◊◊	13
OR8D4	11q24.1	●			✓			✓	CPS-ab	Olfactory transduction	◊◊◊	1
OR10G9	11q24.1	●						✓	CPS-ab	Olfactory transduction	◊◊◊◊	2
OR10G4	11q24.1	●						✓	CPS-ab	Olfactory transduction	◊◊◊◊	2
OR4D5	11q24.1	●						✓	CPS-ab	Olfactory transduction	◊◊◊◊	2
OR10G7	11q24.1	●						✓	CPS-ab	Olfactory transduction	◊◊◊◊	2
OR6T1	11q24.1	●						✓	CPS-ab	Olfactory transduction	◊◊◊◊	2
OR10G8	11q24.1	●						✓	CPS-ab	Olfactory transduction	◊◊◊◊	2
OR10S1	11q24.1	●						✓	CPS-ab	Olfactory transduction	◊◊◊◊	2
OR6M1	11q24.1	●					✓	✓	CPS-ab	Olfactory transduction	◊◊◊◊	1
OR6X1	11q24.1	●					✓	✓	CPS-ab	Olfactory transduction	◊◊◊◊	1
ROBO4	11q24.2	●					✓	✓	CMP-ab	I-sset| fn3| ig	ooooo	39
HEPACAM	11q24.2	●			✓		✓	✓	CMP-ab	I-sset| V-set| ig	ooooo	28
VSIG2	11q24.2	●						✓	CMP-ab	I-sset| V-set| ig	ooooo	67
STT3A	11q24.2	●						✓	CPS-s	Metabolic pathways	◊◊◊◊	1
ROBO3	11q24.2	●					✓	✓	CPS-ab	Axon guidance	◊	15
JAM3	11q25	●●			✓	✓	✓	✓	CMP-ab	C2-set_2| I-sset| V-set| ig	ooooo	17
IGSF9B	11q25	●●					✓	✓	CMP-ab	I-sset| V-set| fn3| ig	ooooo	21
ACAD8	11q25	●●					✓	✓	CPS-s	Metabolic pathways	◊◊◊◊	1
B3GAT1	11q25	●●					✓	✓	CPS-s	Metabolic pathways	◊◊◊◊	1
										**12p13.3-p11.2**		
DDX47	**12p13.1**	●					✓	✓	CMP-ab	DEAD| Helicase_C| ResIII	ooooo	26
CDKN1B	**12p13.1**	●					✓	✓	CPS-ab	Pathways in cancer	◊	9
GRIN2B	**12p13.1**	●		✓	✓		✓	✓	PPI-s	3667	◊◊◊	4
LRP6	**12p13.2**	●						✓	-	-		
PIK3C2G	**12p12.3**	●			✓			✓	CPS-s	Metabolic pathways	◊◊◊◊	1
PLCZ1	**12p12.3**	●			✓			✓	CPS-s	Metabolic pathways	◊◊◊◊	1
BCAT1	**12p12.1**	●				✓	✓	✓	CPS-s	Metabolic pathways	◊◊◊◊	1

KRAS	**12p12.1**	●					✔	✔	CPS-ab	Pathways in cancer	◊	9
CNTN1	12q12	●					✔	✔	CMP-ab	I-sset| V-set| fn3| ig	○○○○○	21
ERBB3	12q13.2	●●					✔	✔	CMP-ab	Furin-like| Pkinase| Pkinase_Tyr| Recep_L_domain	○○○○○	12
MMP19	12q13.2	●●						✔	CMP-ab	Hemopexin| PG_binding_1| Peptidase_M10	○○○○○	37
IL23A	12q13.2	●●					✔	✔	CPS-s	Cytokine-cytokine receptor interaction	◊◊◊	2
CS	12q13.2	●●				✔	✔	✔	CPS-s	Metabolic pathways	◊◊◊◊	1
GLS2	12q13.2	●●					✔	✔	CPS-s	Metabolic pathways	◊◊◊◊	1
DGKA	12q13.2	●●						✔	CPS-s	Metabolic pathways	◊◊◊◊	1
STAT2	12q13.2	●●					✔	✔	CPS-ab	Jak-STAT signaling pathway	◊	10
RAB5B	12q13.2	●●						✔	CPS-ab	Tuberculosis	◊◊◊	5
CDK2	12q13.2	●●						✔	PPI-ab	8317, 2806, 2035, 5898, 4654, 7507, 5583, 898, 5037, 471, 672, 7428, 4332, 1027, 902, 5499, 4088, 5888, 8208, 6502, 80018, 11140, 124930, 192111, 6272, 84811, 5425, 7290, 3398, 932, 988, 1045	◊	17
ATP5B	12q13.3	●●						✔	CPS-s	Metabolic pathways	◊◊◊◊	1
PRIM1	12q13.3	●●						✔	CPS-s	Metabolic pathways	◊◊◊◊	1
SRGAP1	12q14.2	●					✔	✔	CPS-ab	Axon guidance	◊	15
OTOGL	12q21.31	●						✔	CMP-ab	AbfB| C8| TIL| VWD	○○○○○	8
MYF5	12q21.31	●		✔	✔	✔	✔	✔	CMP-ab	Basic| HLH| Myf5	○○○○○	11
ACSS3	12q21.31	●			✔			✔	CPS-s	Metabolic pathways	◊◊◊◊	1
LIN7A	12q21.31	●			✔	✔	✔	✔	CPS-ab	Chaperones modulate interferon Signaling Pathway	◊	11
PRDM4	12q23.3	●			✔		✔	✔	PPI-ab	54855, 4088	◊◊◊	16
ACAD10	12q24.12	●						✔	CMP-ab	Acyl-CoA_dh_1| Acyl-CoA_dh_2| Acyl-CoA_dh_M| Acyl-CoA_dh_N	○○○○○	9
ALDH2	12q24.12	●					✔	✔	CPS-s	Metabolic pathways	◊◊◊◊	1
PTPN11	12q24.13	●						✔	CPS-ab	Jak-STAT signaling pathway	◊	10
VSIG10	12q24.23	●						✔	CMP-ab	I-sset| V-set| ig	○○○○○	67
POLE	12q24.33	●						✔	CPS-s	Metabolic pathways	◊◊◊◊	1
FZD10	12q24.33	●●			✔			✔	CPS-ab	Pathways in cancer	◊◊◊	2
										**13q12**		
CRYL1	**13q12.11**	●						✔	CPS-s	Metabolic pathways	◊◊◊◊	1
FLT3	**13q12.2**	●	✔	✔	✔	✔	✔	✔	CPS-s	Cytokine-cytokine receptor interaction	◊◊◊	2
PRHOXNB	**13q12.2**	●					✔	✔	CPS-s	Metabolic pathways	◊◊◊◊	1
POLR1D	**13q12.2**	●					✔	✔	CPS-s	Metabolic pathways	◊◊◊◊	1
PDX1	**13q12.2**	●					✔	✔	CPS-s	Type II diabetes mellitus	◊◊	4
CDX2	**13q12.2**	●					✔	✔	MIR-ab	MI0000270	◊◊	1
KATNAL1	**13q12.3**	●						✔	CMP-ab	AAA| AAA_2| RuvB_N| Vps4_C	○○○○○	11
FLT1	**13q12.3**	●						✔	CPS-s	Cytokine-cytokine receptor interaction	◊◊◊	2
ALOX5AP	**13q12.3**	●	✔	✔	✔	✔	✔	✔	CPS-ab	Eicosanoid Metabolism	◊	5
KL	**13q13.1**	-										
POSTN	13q13.3	●		✔	✔	✔	✔	✔	CMP-ab	Fasciclin	○○○○	30
KBTBD6	13q14.11	●						✔	CMP-ab	BACK| BTB| Kelch_1	○○○○○	50
KBTBD7	13q14.11	●						✔	CMP-ab	BACK| BTB| Kelch_1	○○○○○	50
DGKH	13q14.11	●						✔	CPS-s	Metabolic pathways	◊◊◊◊	1
ALG11	13q14.3	●●					✔	✔	CPS-s	Metabolic pathways	◊◊◊◊	1
PCDH9	13q21.32	●			✔		✔	✔	CMP-ab	Cadherin| Cadherin_2| Protocadherin	○○○○○	20
EDNRB	13q22.3	●		✔	✔		✔	✔	CMP-ab	7TM_GPCR_Srsx| 7TM_GPCR_Srx| 7tm_1	○○○○○	8
FARP1	13q32.2	●	✔	✔	✔	✔	✔	✔	CMP-ab	FERM_C| FERM_M| FERM_N	○○○○○	2
IPO5	13q32.2	●					✔	✔	PPI-ab	5897, 3021, 1778, 5108, 54606	◊◊	31
IRS2	13q34	●●		✔	✔		✔	✔	CMP-s	3667 IRS| PH	●●●	1
RAB20	13q34	●			✔		✔	✔	CMP-ab	Arf| Miro| Ras	○○○○○	24
COL4A2	13q34	●●	✔	✔	✔	✔	✔	✔	CPS-s	ECM-receptor interaction	◊	1
COL4A1	13q34	●●			✔		✔	✔	CPS-ab	Pathways in cancer	◊◊◊	2
ING1	13q34	●						✔	PPI-ab	10971, 6601	◊	457
NID2	14q22.1	●			✔		✔	✔	CMP-s	4040 Ldl_recept_b	●●●	3
PTGER2	14q22.1	●						✔	CMP-ab	7TM_GPCR_Srsx| 7TM_GPCR_Srx| 7tm_1	○○○○○	65
FRMD6	14q22.1	●					✔	✔	CMP-ab	FERM_C| FERM_M| FERM_N	○○○○○	18
PRKCH	14q23.1	●			✔	✔	✔	✔	CMP-ab	C1_1| Pkinase| Pkinase_C| Pkinase_Tyr	○○○○○	13
HIF1A	14q23.2	●					✔	✔	CPS-s	Hypoxia-Inducible Factor in the Cardiovascular System	◊◊	2
DYNC1H1	14q32.31	●		✔	✔	✔	✔	✔	CPS-s	Phagosome	◊	3
HSP90AA1	14q32.31	●			✔		✔	✔	PPI-s	4846	◊◊◊	2
CDC42BPB	14q32.32	●			✔		✔	✔	CMP-ab	C1_1| Pkinase| Pkinase_C| Pkinase_Tyr	○○○○○	13
TRAF3	14q32.32	●					✔	✔	CPS-ab	CD40L Signaling Pathway	◊◊	7
EIF5	14q32.32	●					✔	✔	CPS-ab	Regulation of eIF2	◊	14
CKB	14q32.33	●						✔	CPS-s	Metabolic pathways	◊◊◊◊	1

SPINT1	15q15.1	●						✔	CMP-s	4040 Ldl_recept_a	●●	5
IVD	15q15.1	●				✔	✔	✔	CPS-s	Metabolic pathways	◊◊◊◊	1
PLCB2	15q15.1	●					✔	✔	CPS-s	Metabolic pathways	◊◊◊◊	1
EIF2AK4	15q15.1	●						✔	CPS-ab	Regulation of eIF2	◊	14
PAK6	15q15.1	●					✔	✔	CPS-ab	Renal cell carcinoma	◊	13
RAD51	15q15.1	●						✔	PPI-s	3667	◊◊◊◊	6
CASC5	15q15.1	●					✔	✔	PPI-ab	5499, 79003, 79980	◊	251
SEMA6D	15q21.1	●	✔	✔	✔	✔	✔	✔	CPS-ab	Axon guidance	◊◊◊	2
LCTL	15q22.31	●			✔				CMP-s	9365 Glyco_hydro_1	●	8
RASL12	15q22.31	●●	✔	✔	✔	✔	✔	✔	CMP-ab	Arf| Miro| Ras	○○○○○	24
IGDCC4	15q22.31	●●						✔	CMP-ab	I-sset| fn3| ig	○○○○○	76
IGDCC3	15q22.31	●●						✔	CMP-ab	I-sset| V-set| fn3| ig	○○○○○	46
PLEKHO2	15q22.31	●●					✔	✔	CPS-s	Cytokine-cytokine receptor interaction	◊◊◊	2
MTFMT	15q22.31	●●				✔	✔	✔	CPS-ab	One carbon pool by folate	◊◊◊	1
SMAD3	15q22.33	●	✔	✔	✔	✔	✔	✔	PPI-s	3667	◊◊◊	3
THSD4	15q23	●	✔	✔	✔	✔	✔	✔	CMP-ab	ADAM_spacer1| TSP_1	○○○○○	5
PSTPIP1	15q24.3	●					✔	✔	CMP-ab	FCH| SH3_1| SH3_2	○○○○○	36
ADAMTS7	15q25.1	●●	✔	✔	✔	✔	✔	✔	CMP-ab	ADAM_spacer1| Pep_M12B_propep| Reprolysin| TSP_1	○○○○○	1
PSMA4	15q25.1	●●	✔	✔	✔	✔	✔	✔	CMP-ab	Proteasome| Proteasome_A_N	○○○○○	6
ACSBG1	15q25.1	●●					✔	✔	CPS-s	Metabolic pathways	◊◊◊◊	1
FAH	15q25.1	●	✔	✔	✔	✔	✔	✔	CPS-s	Metabolic pathways	◊◊◊◊	1
IDH3A	15q25.1	●●						✔	CPS-s	Metabolic pathways	◊◊◊◊	1
MTHFS	15q25.1	●						✔	CPS-s	Metabolic pathways	◊◊◊◊	1
ARNT2	15q25.1	●					✔	✔	CPS-ab	Pathways in cancer	◊	9
WDR61	15q25.1	●●			✔		✔	✔	CPS-ab	RNA degradation	◊◊	6
										**15q26**		
ACAN	**15q26.1**	●	✔	✔	✔	✔	✔	✔	CMP-ab	EGF| Lectin_C| Sushi| V-set| Xlink	○○○○○	10
POLG	**15q26.1**	●						✔	CPS-s	Metabolic pathways	◊◊◊◊	1
MEF2A	**15q26.3**	-										
GRIN2A	16p13.2	●●●			✔				CMP-ab	ANF_receptor| Lig_chan| Lig_chan-Glu_bd| NMDAR2_C| SBP_bac_3	○○○○○	2
PMM2	16p13.2	●●●						✔	CPS-s	Metabolic pathways	◊◊◊◊	1
ABAT	16p13.2	●●●						✔	CPS-s	Metabolic pathways	◊◊◊◊	1
MMP15	16q13	●●						✔	CMP-s	4314 Hemopexin| PG_binding_1| Peptidase_M10	●	17
CNGB1	16q13	●●						✔	CPS-ab	Olfactory transduction	◊◊◊◊	2
GOT2	16q21	●●						✔	CPS-s	Metabolic pathways	◊◊◊◊	1
CNOT1	16q21	●●			✔		✔	✔	CPS-ab	RNA degradation	◊◊	6
GINS3	16q21	●●			✔		✔	✔	PPI-ab	5685, 9837, 4174, 57001, 51659	◊◊◊	46
ADAMTS18	16q23.1	●			✔			✔	CMP-ab	ADAM_spacer1| Pep_M12B_propep| Reprolysin| TSP_1	○○○○○	5
MAF	16q23.1	●●			✔				CMP-ab	Maf_N| bZIP_Maf	○○○○○	27
GAN	16q23.2	●						✔	CMP-ab	BACK| BTB| Kelch_1	○○○○○	50
SDR42E1	16q23.3	●			✔		✔	✔	CMP-ab	3Beta_HSD| Epimerase| NAD_binding_4| Polysacc_synt_2| RmlD_sub_bind	○○○○○	1
PLCG2	16q23.3	●	✔	✔	✔	✔	✔	✔	CPS-s	Metabolic pathways	◊◊◊◊	1
HSD17B2	16q23.3	●					✔	✔	CPS-s	Metabolic pathways	◊◊◊◊	1
MPHOSPH6	16q23.3	●●●			✔			✔	CPS-ab	RNA degradation	◊◊	6
COX4I1	16q24.1	●			✔	✔	✔	✔	CPS-s	Metabolic pathways	◊◊◊◊	1
DHX33	17p13.2	●●					✔	✔	CMP-ab	DEAD| DUF1605| HA2| Helicase_C	○○○○○	9
PIK3R5	17p13.1	●			✔		✔	✔	CPS-s	Type II diabetes mellitus	◊	1
NTN1	17p13.1	●	✔	✔	✔	✔	✔	✔	CPS-ab	Axon guidance	◊◊◊	2
STX8	17p13.1	●	✔	✔	✔	✔	✔	✔	CPS-ab	SNARE interactions in vesicular transport	◊	7
ARHGAP44	17p12	●●	✔	✔	✔	✔	✔	✔	PPI-ab	5879, 51155	◊	97
										**17q11.2-q12**		
CORO6	**17q11.2**	●				✔	✔	✔	CMP-ab	DUF1899| DUF1900| WD40	○○○○○	6
CCL2	**17q12**	-										
KLHL11	17q21.2	●						✔	CMP-ab	BACK| BTB| Kelch_1	○○○○○	50
KLHL10	17q21.2	●						✔	CMP-ab	BACK| BTB| Kelch_1| Kelch_2	○○○○○	22
ACLY	17q21.2	●						✔	CPS-s	Metabolic pathways	◊◊◊◊	1
EIF1	17q21.2	●			✔		✔	✔	CPS-s	VEGF, Hypoxia, and Angiogenesis	◊	4
ARL4D	17q21.31	●			✔	✔	✔	✔	CMP-ab	Arf| Gtr1_RagA| Miro| Ras| SRPRB	○○○○○	4
RND2	17q21.31	●					✔	✔	CMP-ab	Arf| Miro| Ras	○○○○○	30
DHX8	17q21.31	●					✔	✔	CMP-ab	DEAD| DUF1605| HA2| Helicase_C	○○○○○	9
AARSD1	17q21.31	●						✔	CMP-ab	tRNA-synt_2c| tRNA_SAD	○○○○○	71
AOC3	17q21.31	●						✔	CPS-s	Metabolic pathways	◊◊◊◊	1
AOC2	17q21.31	●						✔	CPS-s	Metabolic pathways	◊◊◊◊	1
G6PC	17q21.31	●						✔	CPS-s	Metabolic pathways	◊◊◊◊	1
BRCA1	17q21.31	●					✔	✔	CPS-ab	BRCA1-dependent Ub-ligase activity	◊◊	6
WNT9B	17q21.32	●				✔	✔	✔	CPS-ab	Pathways in cancer	◊	9

WNT3	17q21.32	●					✔	✔	CPS-ab	Pathways in cancer	◊	9
GOSR2	17q21.32	●	✔	✔	✔	✔	✔	✔	CPS-ab	SNARE interactions in vesicular transport	◊	7
ITGB3	17q21.32	●						✔	PPI-s	948	◊◊◊	14
NSF	17q21.32	●					✔	✔	PPI-ab	26227, 10228, 8666, 9568, 3320, 3326, 6950, 1936, 3187, 10963, 3857, 5286, 10652, 5870, 4967, 5708, 2904, 6804, 65263, 255738, 1857, 5707, 80227, 9482, 7419, 6632, 6229, 6160, 3192, 55342, 23344, 51493	◊◊◊	27
NGFR	17q21.33	●						✔	CPS-s	Cytokine-cytokine receptor interaction	◊◊◊	2
XYLT2	17q21.33	●						✔	CPS-s	Metabolic pathways	◊◊◊◊	1
ITGA3	17q21.33	●					✔	✔	CPS-ab	Pathways in cancer	◊	9
PHB	17q21.33	●						✔	PPI-s	3667	◊◊◊◊	10
COL1A1	17q21.33	●						✔	PPI-s	948	◊	20
GALR2	17q25.1	●●					✔	✔	CMP-ab	7TM_GPCR_Srsx| 7TM_GPCR_Srw| 7tm_1	○○○○○	73
GRIN2C	17q25.1	●						✔	CMP-ab	ANF_receptor| Lig_chan| Lig_chan-Glu_bd| NMDAR2_C| SBP_bac_3	○○○○○	5
RECQL5	17q25.1	●●						✔	CMP-ab	DEAD| Helicase_C| ResIII	○○○○○	54
ITGB4	17q25.1	●●					✔	✔	CMP-ab	EGF_2| Integrin_B_tail| Integrin_beta	○○○○○	15
GGA3	17q25.1	●				✔	✔	✔	CMP-ab	GAT| VHS	○○○○○	15
CASKIN2	17q25.1	●●					✔	✔	CMP-ab	SAM_1| SAM_2| SH3_2	○○○○○	37
TRIM47	17q25.1	●●					✔	✔	CMP-ab	SPRY| zf-B_box| zf-C3HC4	○○○○○	33
TRIM65	17q25.1	●●				✔	✔	✔	CMP-ab	SPRY| zf-B_box| zf-C3HC4	○○○○○	10
GALK1	17q25.1	●●					✔	✔	CPS-s	Metabolic pathways	◊◊◊◊	1
ACOX1	17q25.1	●●	✔	✔	✔	✔	✔	✔	CPS-s	Metabolic pathways	◊◊◊◊	1
NT5C	17q25.1	●					✔	✔	CPS-s	Metabolic pathways	◊◊◊◊	1
SPHK1	17q25.1	●●						✔	CPS-s	Metabolic pathways	◊◊◊◊	1
AANAT	17q25.1	●						✔	CPS-s	Metabolic pathways	◊◊◊◊	1
ATP5H	17q25.1	●						✔	CPS-s	Metabolic pathways	◊◊◊◊	1
H3F3B	17q25.1	●●					✔	✔	CPS-ab	Systemic lupus erythematosus	◊◊	3
GRB2	17q25.1	●			✔	✔	✔	✔	PPI-s	3667	◊◊◊◊	1
ICT1	17q25.1	●						✔	PPI-ab	10845, 7428, 5428, 7327, 5300, 2632, 23395, 54995	◊	172
RPTOR	17q25.3	●	✔	✔	✔	✔	✔	✔	CPS-s	Insulin signaling pathway	◊	1
BAIAP2	17q25.3	●					✔	✔	PPI-ab	575, 8825, 5879, 55740, 23189, 8936	◊◊	36
LPIN2	18p11.31	●	✔	✔	✔	✔	✔	✔	CMP-ab	LNS2| Lipin_N	○○○○○	68
LAMA1	18p11.31	●			✔				CPS-ab	Pathways in cancer	◊◊◊	2
PTPRM	18p11.23	●	✔	✔	✔	✔	✔	✔	CPS-ab	Adherens junction	◊◊◊	1
HRH4	18q11.2	●					✔	✔	CMP-s	1524 7TM_GPCR_Srsx| 7tm_1	●	7
SNRPD1	18q11.2	●						✔	CPS-ab	Systemic lupus erythematosus	◊◊◊	3
GATA6	18q11.2	●			✔		✔	✔	MIR-ab	MI0000270	◊◊	1
DSC2	18q12.1	●						✔	CMP-ab	Cadherin| Cadherin_C| Cadherin_pro	○○○○○	44
DSC1	18q12.1	●						✔	CMP-ab	Cadherin| Cadherin_C| Cadherin_pro	○○○○○	44
DSC3	18q12.1	●		✔	✔			✔	CMP-ab	Cadherin| Cadherin_C| Cadherin_pro	○○○○○	16
CDH2	18q12.1	●			✔				PPI-ab	2898, 2904, 165904, 1501, 5793	◊◊	6
FECH	18q21.31	●			✔			✔	CPS-s	Metabolic pathways	◊◊◊◊	1
NETO1	18q22.3	●		✔	✔	✔	✔	✔	CMP-s	4040 Ldl_recept_a	●	4
KCNG2	18q23	●						✔	CMP-ab	Ion_trans| Ion_trans_2| K_tetra	○○○○○	56
TMPRSS9	19p13.3	●					✔	✔	CMP-s	4040 Ldl_recept_a	●●	4
DIRAS1	19p13.3	●						✔	CMP-ab	Arf| Miro| Ras	○○○○○	55
LINGO3	19p13.3	●				✔	✔	✔	CMP-ab	I-sset| V-set| ig	○○○○○	14
OAZ1	19p13.3	●				✔	✔	✔	CMP-ab	ODC_AZ	○○○○	39
AMH	19p13.3	●				✔	✔	✔	CPS-s	Cytokine-cytokine receptor interaction	◊◊◊	2
										**19p13.2**		
RDH8	**19p13.2**	●			✔		✔	✔	CPS-s	Metabolic pathways	◊◊◊◊	1
DNMT1	**19p13.2**	●					✔	✔	CPS-s	Metabolic pathways	◊◊◊◊	1
TYK2	**19p13.2**	●						✔	CPS-ab	Jak-STAT signaling pathway	◊	10
UQCRFS1	19q12	●●●						✔	CPS-s	Metabolic pathways	◊◊◊◊	1
CCNE1	19q12	●●●					✔	✔	CPS-ab	Pathways in cancer	◊	9
CADM4	19q13.31	●						✔	CMP-ab	C2-set_2| I-sset| V-set| ig	○○○○○	42
PLAUR	19q13.31	●						✔	PPI-ab	3827, 3688, 3482, 7297, 6612, 3689, 3693, 6613	◊◊	86
BMP2	20p12.3	●●		✔	✔			✔	CPS-s	Cytokine-cytokine receptor interaction	◊◊◊	2
CRLS1	20p12.3	●●						✔	CPS-s	Metabolic pathways	◊◊◊◊	1
JAG1	20p12.2	●					✔	✔	CMP-ab	EGF| EGF_2| EGF_CA	○○○○○	58
SSTR4	20p11.21	●						✔	CMP-ab	7TM_GPCR_Srsx| 7TM_GPCR_Srv| 7TM_GPCR_Srx| 7tm_1	○○○○○	28
PYGB	20p11.21	●●	✔	✔	✔	✔	✔	✔	CPS-s	Insulin signaling pathway	◊	1
ACSS1	20p11.21	●●					✔	✔	CPS-s	Metabolic pathways	◊◊◊◊	1
NANP	20p11.21	●●						✔	CPS-s	Metabolic pathways	◊◊◊◊	1
EPB41L1	20q11.23	●					✔	✔	CMP-ab	4_1_CTD| FA| FERM_C| FERM_M|	○○○○○	1

										FERM_N| SAB		
MAFB	20q12	●			✔				CMP-ab	Maf_N| bZIP_Maf	○○○○○	27
KCNB1	20q13.13	●						✔	CMP-ab	Ion_trans| Ion_trans_2| K_tetra	○○○○○	56
KCNG1	20q13.13	●						✔	CMP-ab	Ion_trans| Ion_trans_2| K_tetra	○○○○○	56
DPM1	20q13.13	●						✔	CPS-s	Metabolic pathways	◊◊◊◊	1
ARFGEF2	20q13.13	●		✔	✔	✔	✔	✔	CPS-ab	ADP-sRibosylation Factor	◊◊◊	3
NFATC2	20q13.2	●	✔	✔	✔	✔	✔	✔	CPS-ab	Axon guidance	◊◊◊	2
NPBWR2	20q13.33	●			✔	✔	✔	✔	CMP-ab	7TM_GPCR_Srsx| 7TM_GPCR_Srv| 7tm_1	○○○○○	26
OPRL1	20q13.33	●	✔	✔	✔	✔	✔	✔	CMP-ab	7TM_GPCR_Srsx| 7TM_GPCR_Srv| 7TM_GPCR_Srx| 7tm_1	○○○○○	3
ARFRP1	20q13.33	●						✔	CMP-ab	Arf| Gtr1_RagA| Miro| Ras| SRPRB	○○○○○	12
CDH4	20q13.33	●●					✔	✔	CMP-ab	Cadherin| Cadherin_C| Cadherin_pro	○○○○○	44
TNFRSF6B	20q13.33	●						✔	CPS-s	Cytokine-cytokine receptor interaction	◊◊◊	2
UCKL1	20q13.33	●					✔	✔	CPS-s	Metabolic pathways	◊◊◊◊	1
CBR3	21q22.12	●						✔	CPS-s	Metabolic pathways	◊◊◊◊	1
SIM2	21q22.13	●●		✔	✔		✔	✔	CMP-ab	HLH| PAS| PAS_3	○○○○○	25
HLCS	21q22.13	●●			✔		✔	✔	CPS-s	Metabolic pathways	◊◊◊◊	1
PIGP	21q22.13	●●						✔	CPS-s	Metabolic pathways	◊◊◊◊	1
ADARB1	21q22.3	●●	✔	✔	✔	✔	✔	✔	CMP-ab	A_deamin| dsrm	○○○○○	4
ITGB2	21q22.3	●●					✔	✔	CPS-s	Phagosome	◊	3
COMT	22q11.21	●					✔	✔	CPS-s	Metabolic pathways	◊◊◊◊	1
RANBP1	22q11.21	●						✔	PPI-ab	2035, 1104, 7428, 7327, 22918, 8793, 23043, 7189	◊	140
LRP5L	22q11.23	●●●			✔				CMP-s	4040 Ldl_recept_b	●●●	2
SEZ6L	22q12.1	●●●	✔	✔	✔	✔	✔	✔	CMP-ab	CUB| Sushi	○○○○○	1
TMPRSS6	22q12.3	●					✔	✔	CMP-s	4040 Ldl_recept_a	●	12
RASD2	22q12.3	●						✔	CMP-ab	Arf| GTP_EFTU| Miro| Ras	○○○○○	30
TOM1	22q12.3	●				✔	✔	✔	CMP-ab	GAT| VHS	○○○○○	15
CSF2RB	22q12.3	●						✔	CPS-s	Cytokine-cytokine receptor interaction	◊◊◊	2
IL2RB	22q12.3	●					✔	✔	CPS-s	Cytokine-cytokine receptor interaction	◊◊◊	2
MPST	22q12.3	●					✔	✔	CPS-s	Metabolic pathways	◊◊◊◊	1
TST	22q12.3	●					✔	✔	CPS-s	Metabolic pathways	◊◊◊◊	1
SSTR3	22q13.1	●		✔	✔	✔	✔	✔	CMP-ab	7TM_GPCR_Srsx| 7TM_GPCR_Srv| 7TM_GPCR_Srx| 7tm_1	○○○○○	3

Genes in previously associated loci are shaded in orange. Genes and loci in bold have been previously associated with the disease. Genes underlined are the WTCCC candidates. Key to genetic support column: HS ●●●●, MHS-●●●, MWS-●●, WS-●, no support -. Method: ab- *ab initio*, s- *seeded*. Common biological support column depends on method. For CMP-s-s-s, common gene and common domain are listed. For CMP-s-ab, only the common domain. For CPS-s-s, the common pathway is listed. For PPI-s-s, the Entrez gene ID of the gene(s) are listed. For MIR-s-s, the common miRNA ID is listed. For CRT, the common oRegAnno ID is listed. *Gentrepid* scoring: CMP-s-ab: ○○○○○- log $\chi^2 \geq 2.5$, ○○○○- $2 \leq \log \chi^2 < 2.5$, ○○○- $1.5 \leq \log \chi^2 < 2$, ○○- $1 \leq \log \chi^2 < 1.5$, ○- $\log \chi^2 < 1$. CMP-s-s-s: ●●●● - Sc > 0.7, ●●● - Sc > 0.6, ●● - Sc > 0.5, ●-Sc > 0.4. Other: ◊◊◊◊- $p< 0.005$, ◊◊◊ - $p< 0.01$, ◊◊ - $p < 0.025$, ◊- $p< 0.05$. Rank represents ranking score in prioritization of gene in specific set and search space and module, not overall ranking.

Supp. Table S2. Significantly enriched pathways for the CAD phenotype

	Pathway	Resident			Nearest			Adjacent			0.1Mbp			0.5Mbp			1Mbp		
		n	***p***	***r***	***n***	***p***	***r***	***n***	***p***	***r***	***n***	***p***	***r***	***n***	***P***	***r***	***n***	***p***	***r***
HS																			
Ab initio																			
	Melanoma	-	-	-	-	-	-	-	-	-	-	-	-	-	-	-	2	**0.0063**	1
MHS																			
Ab initio	**Steps in the Glycosylation of Mammalian N-linked Oligosaccharides**	-	-	-	-	-	-	2	**0.0003**	1	-	-	-	2	**0.0005**	1	2	**0.0024**	1
	Pathways in cancer	-	-	-	-	-	-	2	0.1076	2	-	-	-	3	**0.0391**	7	4	0.0734	7
	Cyclins and Cell Cycle Regulation	-	-	-	-	-	-	-	-	-	-	-	-	2	**0.0013**	2	2	**0.0056**	2
	Cell Cycle: G1/S Check Point	-	-	-	-	-	-	-	-	-	-	-	-	2	**0.0019**	3	2	**0.0082**	3
	p53 signalling pathway	-	-	-	-	-	-	-	-	-	-	-	-	2	**0.0110**	4	2	**0.0438**	4
	Small cell lung cancer	-	-	-	-	-	-	-	-	-	-	-	-	2	**0.0169**	5	2	0.0652	6
	Cell cycle	-	-	-	-	-	-	-	-	-	-	-	-	2	**0.0342**	6	2	0.1239	9
	Melanoma	-	-	-	-	-	-	-	-	-	-	-	-	-	-	-	2	**0.0461**	5
MWS																			
Seeded																			
	**Insulin signalling pathway**	2	**0.0192**	1	4	**0.0003**	1	4	**0.0177**	3	2	0.1473	2	4	0.3014	8	5	0.6130	20
	**PPAR signalling pathway**	1	0.1074	3	1	0.1567	13	2	0.0947	7	2	**0.0473**	1	3	0.1221	2	4	0.1685	3
	Aldosterone-regulated sodium reabsorption	-	-	-	2	**0.0043**	2	2	**0.0374**	4	-	-	-	1	0.5179	13	2	0.3661	10
	Type II diabetes mellitus	-	-	-	2	**0.0056**	3	3	**0.0050**	1	-	-	-	1	0.5658	16	3	0.1779	4
	Adipocytokine signalling pathway	-	-	-	2	**0.0114**	4	3	**0.0137**	2	-	-	-	2	0.3327	9	5	0.0577	2
	IL 4 signalling pathway	-	-	-	1	**0.0259**	5	1	0.0776	6	-	-	-	-	-	-	1	0.2878	7
	Neurotrophin signalling pathway	-	-	-	2	**0.0358**	6	2	0.2376	14	1	0.4694	4	2	1.0000	21	4	0.7948	25
	Insulin Signalling Pathway	-	-	-	1	**0.0490**	7	1	0.1430	10	-	-	-	-	-	-	1	0.4772	14
	IGF-1 Signalling Pathway	-	-	-	1	**0.0490**	7	1	0.1430	10	-	-	-	-	-	-	1	0.4772	14
Ab initio																			
	**Axon guidance**	2	**0.0169**	1	2	**0.0368**	6	2	0.2430	21	2	0.1322	4	3	0.4869	26	4	0.7978	73
	**Insulin signalling pathway**	2	**0.0192**	2	4	**0.0003**	1	4	**0.0177**	8	2	0.1473	5	4	0.3014	17	5	0.6130	54
	Aldosterone-regulated sodium reabsorption	-	-	-	2	**0.0043**	2	2	**0.0374**	11	-	-	-	-	-	-	2	0.3661	41
	Type II diabetes mellitus	-	-	-	2	**0.0056**	3	3	**0.0050**	2	-	-	-	-	-	-	3	0.1779	27
	Adipocytokine signalling pathway	-	-	-	2	**0.0114**	4	3	**0.0137**	5	-	-	-	2	0.3327	19	5	0.0577	11
	Neurotrophin signalling pathway	-	-	-	2	**0.0358**	5	2	0.2376	20	-	-	-	2	1.0000	39	4	0.7948	72
	**Angiotensin-converting enzyme 2 regulates heart function**	-	-	-	-	-	-	2	**0.0039**	1	-	-	-	-	-	-	2	0.0574	10
	**Steps in the Glycosylation of Mammalian N-linked Oligosaccharides**	-	-	-	-	-	-	2	**0.0052**	3	-	-	-	2	**0.0265**	1	2	0.0743	15
	Basal cell carcinoma	-	-	-	-	-	-	3	**0.0074**	4	-	-	-	-	-	-	3	0.2330	32
	RNA degradation	-	-	-	-	-	-	3	**0.0148**	6	-	-	-	2	0.3453	21	4	0.1685	25
	Cell Cycle: G1/S Check Point	-	-	-	-	-	-	2	**0.0175**	7	-	-	-	2	0.0827	4	4	**0.0095**	4
	Small cell lung cancer	-	-	-	-	-	-	3	**0.0238**	9	-	-	-	2	0.6580	30	5	0.1869	29
	Pathways in cancer	-	-	-	-	-	-	6	**0.0297**	10	-	-	-	5	1.0000	39	11	0.6207	55
	N-Glycan biosynthesis	-	-	-	-	-	-	2	**0.0495**	12	-	-	-	2	0.2053	13	2	0.6606	57
	PPAR signalling pathway	-	-	-	-	-	-	2	0.0947	14	2	**0.0473**	3	3	0.1221	5	4	0.1685	25

Pathway	Resident			Nearest			Adjacent			0.1Mbp			0.5Mbp			1Mbp		
	n	***p***	***r***	***n***	***p***	***r***	***n***	***p***	***r***	***n***	***p***	***r***	***n***	***P***	***r***	***n***	***p***	***r***
**One carbon pool by folate**	-	-	-	-	-	-	-	-	-	2	**0.0034**	1	2	**0.0374**	2	2	0.1021	19
Aminoacyl-tRNA biosynthesis	-	-	-	-	-	-	-	-	-	2	**0.0171**	2	2	0.1559	8	3	0.1272	21
Cyclins and Cell Cycle Regulation	-	-	-	-	-	-	-	-	-	-	-	-	2	0.0585	3	3	**0.0312**	7
Amino sugar and nucleotide sugar metabolism	-	-	-	-	-	-	-	-	-	-	-	-	2	0.1990	12	5	**0.0146**	5
**Olfactory transduction**	-	-	-	-	-	-	-	-	-	-	-	-	-	-	-	2	**0.0010**	1
E2F1 Destruction Pathway	-	-	-	-	-	-	-	-	-	-	-	-	-	-	-	3	**0.0028**	2
Regulation of p27 Phosphorylation during Cell Cycle Progression	-	-	-	-	-	-	-	-	-	-	-	-	-	-	-	3	**0.0063**	3
Cyclin E Destruction Pathway	-	-	-	-	-	-	-	-	-	-	-	-	-	-	-	2	**0.0287**	6
WS																		
Seeded																		
**ECM-receptor interaction**	3	**0.0470**	1	3	0.1216	4	6	0.1387	6	4	0.1443	3	9	0.2979	12	15	0.3485	11
**Insulin signalling pathway**	3	0.1441	5	5	**0.0471**	1	8	0.2514	13	4	0.5723	14	10	1.0000	29	18	0.8052	31
**Type II diabetes mellitus**	-	-	-	2	0.1500	7	5	**0.0339**	1	1	1.0000	22	7	0.0903	5	13	**0.0196**	4
Insulin Signalling Pathway	-	-	-	1	0.2615	11	3	**0.0430**	2	1	0.3981	8	1	1.0000	29	4	0.5271	19
IGF-1 Signalling Pathway	-	-	-	1	0.2615	11	3	**0.0430**	2	1	0.3981	8	1	1.0000	29	4	0.5271	19
**Metabolic pathways**	14	0.3075	8	17	0.7819	20	36	0.3003	16	24	0.5912	16	55	**0.0001**	1	100	**0.0000**	1
Cytokine-cytokine receptor interaction	1	0.5213	9	4	0.7983	21	7	0.3326	17	3	0.2201	4	16	0.2950	11	24	**0.0061**	2
Hypoxia-Inducible Factor in the Cardiovascular System	-	-	-	-	-	-	1	0.4396	19	-	-	-	4	**0.0227**	2	6	**0.0129**	3
Phagosome	1	1.0000	13	2	1.0000	22	6	0.8275	29	2	0.5872	15	5	**0.0424**	3	15	0.1551	8
VEGF, Hypoxia, and Angiogenesis	-	-	-	-	-	-	1	1.0000	30	-	-	-	5	**0.0494**	4	7	0.0937	6
Ab initio																		
**Adherens junction**	4	**0.0047**	1	4	**0.0193**	3	7	**0.0185**	9	5	**0.0281**	4	9	0.1157	29	11	0.7356	172
**Axon guidance**	5	**0.0074**	2	7	**0.0024**	1	9	0.0616	22	8	**0.0116**	2	16	**0.0432**	15	22	0.3713	95
Vibrio cholerae infection	3	**0.0145**	3	3	**0.0416**	6	3	0.4586	102	3	0.1373	23	5	0.6022	108	7	1.0000	215
Arrhythmogenic right ventricular cardiomyopathy (ARVC)	3	**0.0331**	4	3	0.0890	11	5	0.2041	61	3	0.2587	38	6	0.8241	156	12	0.6148	148
Pathways in cancer	7	**0.0338**	5	9	0.0500	8	24	**0.0014**	2	11	0.2576	37	35	**0.0310**	9	54	0.2205	63
Protein digestion and absorption	3	**0.0404**	6	3	0.1062	15	5	0.2297	68	4	0.1231	22	10	0.1305	33	16	0.1456	40
SNARE interactions in vesicular transport	2	**0.0458**	7	2	0.0933	12	3	0.1539	50	2	0.2115	32	3	0.7515	141	7	0.3403	84
ECM-receptor interaction	3	**0.0470**	8	3	0.1216	18	6	0.1387	41	4	0.1443	26	9	0.2979	61	15	0.3485	86
Platelet Amyloid Precursor Protein Pathway	-	-	-	2	**0.0165**	2	3	**0.0143**	8	2	**0.0426**	7	2	0.2876	59	4	0.1260	36
Acute Myocardial Infarction	-	-	-	2	**0.0325**	4	3	**0.0379**	14	2	0.0811	14	2	0.6607	122	5	0.1902	54
Eicosanoid Metabolism	-	-	-	2	**0.0388**	5	2	0.2015	60	2	0.0957	17	2	0.6813	126	5	0.2277	66
Insulin signalling pathway	3	0.1441	17	5	**0.0471**	7	8	0.2514	72	4	0.5723	58	10	1.0000	164	18	0.8052	190
TGF-beta signalling pathway	-	-	-	3	0.1184	16	7	**0.0390**	16	4	0.1400	24	7	0.6813	125	11	0.8757	208
Type II diabetes mellitus	-	-	-	2	0.1500	22	5	**0.0339**	12	-	-	-	7	0.0903	23	13	**0.0196**	9
**Olfactory transduction**	2	0.5850	40	2	0.1781	25	4	**0.0014**	1	3	**0.0360**	5	6	**8.70E-08**	1	8	**6.17E-17**	2
**ADP-Ribosylation Factor**	-	-	-	-	-	-	4	**0.0032**	3	3	**0.0071**	1	4	**0.0352**	11	4	0.2877	74
Small cell lung cancer	2	0.1942	24	2	0.3442	43	9	**0.0046**	4	3	0.4574	56	11	0.0934	25	16	0.2121	60
Rac 1 cell motility signalling pathway	-	-	-	-	-	-	4	**0.0072**	5	-	-	-	5	**0.0181**	5	6	0.1062	31
Angiotensin-converting enzyme 2 regulates heart function	-	-	-	-	-	-	3	**0.0115**	6	-	-	-	2	0.2585	54	2	0.7064	166

Pathway	Resident			Nearest			Adjacent			0.1Mbp			0.5Mbp			1Mbp		
	n	***p***	***r***	***n***	***p***	***r***	***n***	***p***	***r***	***n***	***p***	***r***	***n***	***P***	***r***	***n***	***p***	***r***
How does salmonella hijack a cell	-	-	-	-	-	-	2	**0.0132**	7	-	-	-	2	**0.0492**	16	2	0.1504	42
Acute myeloid leukaemia	-	-	-	2	0.1960	29	6	**0.0199**	10	2	0.3960	51	8	0.0749	21	13	0.0827	23
Chaperones modulate interferon Signalling Pathway	-	-	-	-	-	-	3	**0.0287**	11	2	0.0673	9	3	0.1516	37	4	0.3097	78
Role of PI3K subunit p85 in regulation of Actin Organization and Cell Migration	-	-	-	-	-	-	2	**0.0343**	13	-	-	-	-	-	-	2	0.3178	81
fMLP induced chemokine gene expression in HMC-1 cells	-	-	-	-	-	-	4	**0.0381**	15	2	0.1940	30	3	0.7409	140	4	1.0000	215
Endometrial cancer	-	-	-	-	-	-	5	**0.0425**	17	2	0.3444	45	6	0.2788	56	8	0.6896	164
Insulin Signalling Pathway	-	-	-	-	-	-	3	**0.0430**	18	-	-	-	-	-	-	4	0.5271	127
IGF-1 Signalling Pathway	-	-	-	-	-	-	3	**0.0430**	18	-	-	-	-	-	-	4	0.5271	127
Bacterial invasion of epithelial cells	-	-	-	-	-	-	6	**0.0454**	19	2	0.6806	66	5	1.0000	164	7	0.3897	100
Valine, leucine and isoleucine biosynthesis	-	-	-	-	-	-	-	-	-	2	**0.0270**	3	2	0.2008	43	2	0.6623	158
Non-small cell lung cancer	2	0.0934	12	2	0.1804	26	4	0.1465	44	4	**0.0391**	6	6	0.2992	62	8	0.8453	199
**Metabolic pathways**	14	0.3075	30	17	0.7819	62	36	0.3003	81	24	0.5912	61	55	**0.0001**	2	100	**4.39E-09**	1
Systemic lupus erythematosus	-	-	-	-	-	-	3	0.4907	106	-	-	-	3	**0.0126**	3	7	**0.0015**	3
Toxoplasmosis	-	-	-	-	-	-	3	0.4897	105	-	-	-	3	**0.0177**	4	12	0.1262	38
BRCA1-dependent Ub-ligase activity	-	-	-	-	-	-	-	-	-	-	-	-	3	**0.0182**	6	3	0.0922	26
Hypoxia-Inducible Factor in the Cardiovascular System	-	-	-	-	-	-	-	-	-	-	-	-	4	**0.0227**	7	6	**0.0129**	8
CD40L Signalling Pathway	-	-	-	-	-	-	2	0.1084	34	-	-	-	4	**0.0227**	7	4	0.2535	69
Tuberculosis	-	-	-	-	-	-	-	-	-	-	-	-	6	**0.0300**	8	13	**0.0061**	5
Ahr Signal Transduction Pathway	-	-	-	-	-	-	-	-	-	-	-	-	2	**0.0311**	10	2	0.0994	28
Pancreatic cancer	2	0.1401	16	2	0.2594	34	5	0.1879	58	4	0.0817	15	10	**0.0381**	12	12	0.4860	123
Renal cell carcinoma	-	-	-	-	-	-	3	0.7481	118	-	-	-	10	**0.0409**	13	14	0.1678	51
Phagosome	-	-	-	2	1.0000	65	6	0.8275	126	2	0.5872	59	5	**0.0424**	14	15	0.1551	46
Signal Dependent Regulation of Myogenesis by Corepressor MITR	-	-	-	-	-	-	-	-	-	-	-	-	2	**0.0492**	16	2	0.1504	42
VEGF, Hypoxia, and Angiogenesis	-	-	-	-	-	-	-	-	-	-	-	-	5	**0.0494**	17	7	0.0937	27
Cytokine-cytokine receptor interaction	-	-	-	4	0.7983	64	7	0.3326	83	3	0.2201	33	16	0.2950	60	24	**0.0061**	4
Retinol metabolism	-	-	-	-	-	-	-	-	-	-	-	-	-	-	-	2	**0.0063**	6
Drug metabolism - cytochrome P450	-	-	-	-	-	-	-	-	-	-	-	-	-	-	-	3	**0.0103**	7
Jak-STAT signalling pathway	-	-	-	-	-	-	3	0.2875	78	-	-	-	8	0.2849	57	13	**0.0355**	10
Metabolism of xenobiotics by cytochrome P450	-	-	-	-	-	-	-	-	-	2	0.6869	67	2	0.1725	41	4	**0.0386**	11
Glutathione metabolism	-	-	-	-	-	-	-	-	-	-	-	-	-	-	-	2	**0.0394**	12
Arachidonic acid metabolism	-	-	-	-	-	-	-	-	-	-	-	-	-	-	-	3	**0.0395**	13
Regulation of eIF2	-	-	-	-	-	-	-	-	-	-	-	-	2	0.1725	40	4	**0.0418**	14

Pathways in bold are those ranked first in the set and mapping. *n* – number of genes in search space that are in the pathway. *p* – the *p*-value showing the statistical significance of the pathway as calculated by the Fishers-test. *r* – rank of pathway based on *p*-value. *p*-values in bold are those that are significant.

Supp. Table S3. CAD CMP *seeded* results

Gene	Seed	Score	Domain	Resident		Near				Adjacent						0.1Mbp		0.5Mbp				1Mbp			
				WS		MWS		WS		MHS		MWS		WS		WS		MWS		WS		MWS		WS	
				n	***m***	***n***	***m***	***n***	***m***	***n***	***m***	***n***	***m***	***n***	***m***	***n***	***m***	***n***	***m***	***n***	***m***	***n***	***m***	***n***	***m***
PLG	*LPA*	0.826	DUF1986|Kringle|Trypsin																					1	1
IRS2	*IRS1*	0.753	IRS|PH			1	1	1	1			1	1	1	1			1	1	1	1	1	1	1	1
LRP5L	*LRP6*	0.675	Ldl_recept_b							1	1	1	1	2	1										
NID2	*LRP6*	0.66	Ldl_recept_b											1	1					1	1			1	1
NID1	*LRP6*	0.599	Ldl_recept_b																	1	1			1	1
SPINT1	*LRP6*	0.585	Ldl_recept_a																					1	1
LDLRAD3	*LRP6*	0.581	Ldl_recept_a																					1	1
TMPRSS9	*LRP6*	0.532	Ldl_recept_a																	1	1			1	1
LRP11	*LRP6*	0.511	Ldl_recept_a	1	1			1	1					1	1	1	1			1	1			1	1
CCR8	*CX3CR1*	0.487	7TM_GPCR_Srsx|7tm_1															3	1	8	2	3	1	8	2
CCR2	*CX3CR1*	0.486	7TM_GPCR_Srsx|7tm_1																					2	1
CCR5	*CX3CR1*	0.486	7TM_GPCR_Srsx|7tm_1																					2	1
HRH4	*CX3CR1*	0.484	7TM_GPCR_Srsx|7tm_1																	1	1			1	1
ABCA12	*ABCA1*	0.479	ABC_tran	1	1			1	1					1	1	1	1			1	1			1	1
CCR1	*CX3CR1*	0.474	7TM_GPCR_Srsx|7tm_1																	2	1			2	1
NETO1	*LRP6*	0.469	Ldl_recept_a					1	1					1	1	1	1			1	1			1	1
CCR3	*CX3CR1*	0.459	7TM_GPCR_Srsx|7tm_1											2	1					2	1			2	1
TMPRSS6	*LRP6*	0.458	Ldl_recept_a																	1	1			1	1
MMP15	*MMP3*	0.456	Hemopexin|PG_binding_1|Peptidase_M10																			1	1	1	1
LCTL	*KL*	0.413	Glyco_hydro_1											2	1										

Domains have Pfam abbreviations. *n* – number of SNPs. *m* – number of clusters formed by SNPs.

Supp. Table S4. CAD CMP *ab initio* results

Domains	*n*	Genes	χ^2_{min}	$\chi^2_{max\_unique}$
MHS				
Resident				
CUB| Sushi	2	*CSMD2, SEZ6L*	514.2362	381046.4
CH	2	*MICAL2, PARVA*	339.679	339.679
Nearest				
CUB| Sushi	2	*CSMD2, SEZ6L*	292.1441	217739.1
CH	2	*MICAL2, PARVA*	192.4017	192.4017
0.1Mbp				
CUB| Sushi	2	*CSMD2, SEZ6L*	235.1991	175865.4
CH	2	*MICAL2, PARVA*	154.6409	154.6409
0.5Mbp				
I-set| V-set| ig	2	*KALRN, MFAP3L*	11.39225	411152.3
MWS				
Resident				
CUB| Sushi	2	*CSMD2, SEZ6L*	155.48	117242.3
Sema	2	*PLXNA2, SEMA3C*	267.5191	267.5191
CH	2	*MICAL2, PARVA*	101.7816	101.7816
Nearest				
Sema	2	*PLXNA2, SEMA3C*	125.1611	125.1611
0.1Mbp				
Arf| Miro| Ras	2	*RAB6A, RASL12*	12.09576	1136041
0.5Mbp				
EGF_2| Integrin_B_tail| Integrin_beta	2	*ITGB2, ITGB4*	98.26503	6.84E+08
Calpain_III| Peptidase_C2	2	*CAPN11, CAPN2, CAPN8*	48.95111	170562
Proteasome| Proteasome_A_N	2	*PSMA4, PSMA5*	72.72159	159724
1Mbp				
EGF_2| Integrin_B_tail| Integrin_b_cyt| Integrin_beta	2	*ITGB2, ITGB5*	58.91449	2.8E+12
EGF_2| Integrin_B_tail| Integrin_beta	3	*ITGB2, ITGB4, ITGB5*	116.6326	8.2E+08
Hemopexin| PG_binding_1| Peptidase_M10	2	*MMP15, MMP19*	21.68982	1.82E+08
7tm_2| DUF3497| GPS	2	*BAI1, CELSR2*	36.95096	34129528
Calpain_III| Peptidase_C2	3	*CAPN2, CAPN9, CAPN11, CAPN8*	57.53464	204486.5
WS				
Resident				
ADAM_spacer1| Pep_M12B_propep| Reprolysin| TSP_1	2	*ADAMTS2, ADAMTS7*	98.23837	1.33E+11
FERM_C| FERM_M| FERM_N	2	*FARP1, FRMD4A*	51.20635	51478796
EGF| Laminin_G_1| Laminin_G_2	2	*CELSR2, EYS*	49.79457	34242798
A_deamin| dsrm	2	*ADARB1, ADARB2*	186.4832	272084.9
ADAM_spacer1| TSP_1	3	*ADAMTS2, THSD4, ADAMTS7*	223.9874	221098.1
Ded_cyto	2	*DOCK4, DOCK8*	205.5275	205.5275
Peptidase_M1	2	*RNPEPL1, ERAP2*	151.2172	151.2172
CH	5	*MICAL2, IQGAP2, PARVA, SYNE1, LIMCH1*	128.0159	128.0159
Sema	3	*PLXNA2, SEMA3C, SEMA6D*	121.4744	121.4744
Nearest				
FA| FERM_C| FERM_M| FERM_N	2	*EPB41, FARP1*	46.49508	6.19E+10
ADAM_spacer1| Pep_M12B_propep| Reprolysin| TSP_1	2	*ADAMTS2, ADAMTS7*	42.81239	6.08E+10
7TM_GPCR_Srsx| 7TM_GPCR_Srv| 7TM_GPCR_Srx| 7tm_1	2	*OPRL1, SSTR3*	30.32761	5.45E+08
7tm_2| DUF3497| GPS	2	*BAI1, CELSR2*	91.83167	80024987
FERM_C| FERM_M| FERM_N	3	*EPB41, FRMD4A, FARP1*	50.87639	52957708
EGF| Laminin_G_1| Laminin_G_2	2	*CELSR2, EYS*	20.72437	15656240
7TM_GPCR_Srsx| 7TM_GPCR_Srx| 7tm_1	3	*EDNRB, SSTR3, OPRL1*	31.72312	687200.8
A_deamin| dsrm	2	*ADARB1, ADARB2*	83.12767	124398.5
ADAM_spacer1| TSP_1	3	*ADAMTS2, THSD4, ADAMTS7*	99.22088	101085.6
Fasciclin	2	*TGFBI, POSTN*	187.6007	187.6007
TSP_1	5	*BAI1, ADAMTS2, RSPO3, ADAMTS7, THSD4*	105.3492	105.3492
Adjacent				
3Beta_HSD| Epimerase| NAD_binding_4| Polysacc_synt_2| RmlD_sub_bind	2	*HSD3B1, SDR42E1*	23.77582	3.55E+15
ANF_receptor| Lig_chan| Lig_chan-Glu_bd| NMDAR2_C| SBP_bac_3	2	*GRIN2A, GRIN2B*	82.88652	1.01E+15
ANF_receptor| Lig_chan| Lig_chan-Glu_bd| SBP_bac_3	3	*GRIK2, GRIN2A, GRIN2B*	24.03611	1.6E+11
ADAM_spacer1| Pep_M12B_propep| Reprolysin| TSP_1	3	*ADAMTS2, ADAMTS7, ADAMTS18*	27.62923	4.34E+10
C2| PI-PLC-X| PI-PLC-Y| efhand_like	2	*PLCG2, PLCZ1*	13.5984	3.79E+10

	Domains	*n*	Genes	χ^2_{min}	$\chi^2_{max\_unique}$
	FA| FERM_C| FERM_M| FERM_N	2	*EPB41, FARP1*	12.24698	1.97E+10
	7TM_GPCR_Srsx| 7TM_GPCR_Srv| 7TM_GPCR_Srx| 7tm_1	3	*OPRD1, SSTR3, OPRL1*	18.79237	3.89E+08
	C8| TIL| VWD	2	*LOC100652946, OTOG*	23.77582	3.16E+08
	IRF-3| MH1| MH2	2	*SMAD3, SMAD5*	17.15232	1.1E+08
	ADAM_spacer1| PLAC| TSP_1	2	*THSD4, ADAMTS18*	18.69186	52800639
	FERM_C| FERM_M| FERM_N	4	*EPB41, FRMD4A, RDX, FARP1*	24.49395	29872872
	7tm_2| DUF3497| GPS	2	*BAI1, CELSR2*	26.52577	25391944
	Cadherin| Cadherin_C| Cadherin_pro	2	*CDH2, DSC3*	26.52577	25301055
	Cadherin| Cadherin_2| Protocadherin	2	*PCDH9, PCDH7*	18.69186	4467453
	7TM_GPCR_Srsx| 7TM_GPCR_Srx| 7tm_1	5	*EDNRB, HTR1A, SSTR3, OPRL1, OPRD1*	23.79386	605686.9
	7TM_GPCR_Srsx| 7TM_GPCR_Srv| 7tm_1	3	*NPBWR2, OPRL1, SSTR3, OPRD1*	18.79237	447777.1
	Maf_N| bZIP_Maf	2	*MAF, MAFB*	82.88652	413522
	DUF3398| Ded_cyto	2	*DOCK8, DOCK7*	36.62425	202623.7
	nlz1	2	*ZNF703, ZNF503*	198.6475	198.6475
0.1Mbp					
	Arf| Gtr1_RagA| Miro| Ras| SRPRB	2	*ARL4D, ARL15*	8.852718	1.56E+11
	FA| FERM_C| FERM_M| FERM_N	2	*EPB41, FARP1*	26.14788	3.69E+10
	ADAM_spacer1| Pep_M12B_propep| Reprolysin| TSP_1	2	*ADAMTS2, ADAMTS7*	23.96221	3.62E+10
	7TM_GPCR_Srsx| 7TM_GPCR_Srv| 7TM_GPCR_Srx| 7tm_1	3	*OPRD1, SSTR3, OPRL1*	40.02228	7.31E+08
	C8| TIL| VWD	2	*LOC100652946, OTOG*	47.92058	5.92E+08
	DUF1899| DUF1900| WD40	2	*CORO2A, CORO6*	72.08963	59749344
	FERM_C| FERM_M| FERM_N	3	*EPB41, FRMD4A, FARP1*	28.03298	31527949
	EGF| Laminin_G_1| Laminin_G_2	2	*CELSR2, EYS*	10.89605	9320817
	DEAD| Helicase_C| ResIII	2	*DDX56, HFM1*	11.08245	4464254
	7TM_GPCR_Srsx| 7TM_GPCR_Srv| 7tm_1	3	*NPBWR2, OPRL1, SSTR3, OPRD1*	40.02228	840157.6
	DUF3398| Ded_cyto	2	*DOCK8, DOCK7*	72.08963	380181.1
	GAT| VHS	2	*TOM1, GGA3*	47.92058	185151.1
	ODC_AZ	2	*OAZ1, OAZ3*	186.1136	186.1136
	BNR	2	*RELN, SORT1*	186.1136	186.1136
	Ded_cyto	3	*DOCK4, DOCK7, DOCK8*	122.3826	122.3826
	Fasciclin	2	*TGFBI, POSTN*	110.0906	110.0906
0.5Mbp					
	4_1_CTD| FA| FERM_C| FERM_M| FERM_N| SAB	2	*EPB41, EPB41L1*	19.86427	4.26E+17
	3Beta_HSD| Epimerase| NAD_binding_4| Polysacc_synt_2| RmlD_sub_bind	2	*HSD3B1, SDR42E1, HSD3B2*	11.3442	1.94E+15
	EGF_CA| G2F| Ldl_recept_b| NIDO| Thyroglobulin_1	2	*NID1, NID2*	43.47533	1.63E+15
	Acyl-CoA_dh_1| Acyl-CoA_dh_2| Acyl-CoA_dh_M| Acyl-CoA_dh_N	2	*IVD, ACAD8*	11.3442	7.93E+11
	FA| FERM_C| FERM_M| FERM_N	3	*EPB41, FARP1, EPB41L1*	14.10048	2.41E+10
	Basic| HLH| Myf5	2	*MYF5, MYOD1*	106.615	8.65E+08
	Furin-like| Pkinase| Pkinase_Tyr| Recep_L_domain	2	*ERBB3, INSRR*	14.64684	5.87E+08
	7TM_GPCR_Srsx| 7TM_GPCR_Srv| 7TM_GPCR_Srx| 7tm_1	3	*OPRD1, SSTR3, OPRL1*	8.003931	2.12E+08
	C8| TIL| VWD	2	*LOC100652946, OTOG*	11.3442	1.72E+08
	EGF_2| Integrin_B_tail| Integrin_beta	2	*ITGB2, ITGB4*	18.29663	1.48E+08
	FERM_C| FERM_M| FERM_N	6	*EPB41, FRMD4A, FRMD6, RDX, EPB41L1, FARP1*	28.20097	36680268
	DUF1899| DUF1900| WD40	2	*CORO2A, CORO6*	18.29663	17378405
	7tm_2| DUF3497| GPS	2	*BAI1, CELSR2*	12.82798	13856991
	7TM_GPCR_Srsx| 7TM_GPCR_Srx| 7tm_1	6	*ADRA2C, EDNRB, CCR1, SSTR3, OPRL1, OPRD1*	15.34863	475970.4
	7TM_GPCR_Srsx| 7TM_GPCR_Srv| 7tm_1	3	*NPBWR2, OPRL1, SSTR3, OPRD1*	8.003931	244360
	Calpain_III| Peptidase_C2	4	*CAPN2, CAPN10, CAPN9, CAPN11, CAPN8*	38.10643	147594.4
	DUF3398| Ded_cyto	2	*DOCK8, DOCK7*	18.29663	110574.8
1Mbp					
	4_1_CTD| FA| FERM_C| FERM_M| FERM_N| SAB	2	*EPB41, EPB41L1*	8.662955	2.22E+17
	PI3K_C2| PI3K_rbd| PI3Ka| PI3_PI4_kinase| PX	2	*PIK3C2A, PIK3C2G*	13.50584	3.54E+15
	EGF_CA| G2F| Ldl_recept_b| NIDO| Thyroglobulin_1	2	*NID1, NID2*	20.83964	8.47E+14
	ANF_receptor| Lig_chan| Lig_chan-Glu_bd| NMDAR2_C| SBP_bac_3	2	*GRIN2B, GRIN2C*	20.83964	2.88E+14
	C2| PI-PLC-X| PI-PLC-Y| PLC-beta_C| efhand_like	2	*PLCB2, PLCB3*	20.83964	1.54E+14
	EGF_2| Integrin_B_tail| Integrin_b_cyt|	4	*ITGB1, ITGB5, ITGB3, ITGB2*	45.45267	2.37E+12

Domains	*n*	Genes	χ^2_{min}	$\chi^2_{max\_unique}$
Integrin_beta				
AbfB| C8| TIL| VWD	2	*OTOGL, OTOG*	39.27833	2.24E+12
Acyl-CoA_dh_1| Acyl-CoA_dh_2| Acyl-CoA_dh_M| Acyl-CoA_dh_N	3	*IVD, ACAD10, ACAD8*	12.17631	9.29E+11
AAA| AAA_2| RuvB_N| Vps4_C	2	*KATNA1, KATNAL1*	11.95861	1.02E+11
C2| PI-PLC-X| PI-PLC-Y| efhand_like	4	*PLCB2, PLCG2, PLCZ1, PLCB3*	12.55249	4.31E+10
FA| FERM_C| FERM_M| FERM_N	4	*EPB41, EPB41L4B, FARP1, EPB41L1*	11.06338	2.23E+10
EGF| Kringle| Trypsin| fn1	2	*HGFAC, PLAT*	9.588936	9.28E+09
Cu_amine_oxid| Cu_amine_oxidN2| Cu_amine_oxidN3	2	*ABP1, AOC3, AOC2*	24.92941	8E+09
EGF_2| Integrin_B_tail| Integrin_beta	5	*ITGB1, ITGB4, ITGB2, ITGB3, ITGB5*	62.32352	4.82E+08
Basic| HLH| Myf5	2	*MYF5, MYOD1*	53.65041	4.51E+08
C8| TIL| VWD	3	*LOC100652946, OTOG, OTOGL*	12.17631	2.02E+08
I-set| Pkinase| Pkinase_Tyr| V-set| ig	2	*FLT1, MYLK, KALRN*	9.343546	53116135
ADAM_spacer1| PLAC| TSP_1	3	*ADAMTSL2, ADAMTS18, THSD4*	9.020443	33759929
FERM_C| FERM_M| FERM_N	7	*EPB41, FRMD4A, FRMD6, RDX, PTPN3, EPB41L1, EPB41L4B, FARP1*	15.60326	25997574
DUF1899| DUF1900| WD40	3	*CORO2A, CORO6, CORO1B*	20.25876	20361007
Cadherin| Cadherin_C| Cadherin_pro	3	*CDH4, DSC2, DSC3, DSC1*	13.89668	16177109
7TM_GPCR_Srv| 7TM_GPCR_Srx| 7tm_1	5	*OPRD1, PTGFR, OPRL1, SSTR3, SSTR4*	10.57606	1496833
7TM_GPCR_Srsx| 7TM_GPCR_Srx| 7tm_1	9	*ADRA2C, PTGER2, EDNRB, CHRM3, CCR1, SSTR3, OPRL1, SSTR4, OPRD1*	15.08068	557655.8
LNS2| Lipin_N	2	*LPIN2, LPIN1*	39.27833	392593.3
7TM_GPCR_Srsx| 7TM_GPCR_Srv| 7tm_1	5	*NPBWR2, HTR2B, OPRL1, SSTR3, SSTR4, OPRD1*	10.57606	353453.9
tRNA-synt_2c| tRNA_SAD	2	*AARS2, AARSD1*	24.92941	287900.7
RNase_PH| RNase_PH_C	2	*EXOSC7, PNPT1*	13.50584	157034.9
Pkinase| Pkinase_C| Pkinase_Tyr	6	*PRKCH, LATS1, RPS6KA4, SGK1, RPS6KB2, CDC42BPB*	8.258661	116488.7

Domains have Pfam abbreviations. Results are filtered on threshold of $\chi^2_{max\_unique} > 10^5$ and $\chi^2_{min} > 7.88$ for multidomain proteins, and $\chi^2_{min} > 100$ for single domain proteins. *n* – number of genes. Genes are those with the homologous domains.

Supp. Table S5. CAD PPI seeded interactions, with scores and ranks

	Candidate gene	Interacting gene	Resident			Nearest			Adjacent			0.1Mbp			0.5Mbp			1Mbp		
			n	*p*	*r*	*n*	*p*	*r*	*n*	*p*	*r*	*n*	*p*	*r*	*n*	*p*	*r*	*n*	*p*	*r*
MHS																				
	LIMK1	*ABCA1*	-	-	-	-	-	-	-	-	-	-	-	-	1	0.1167	1	1	0.2100	1
MWS																				
	TP53BP2	*IRS1*	-	-	-	-	-	-	-	-	-	-	-	-	-	-	-	1	0.5876	1
	HSP90AB1	*IRS1*	-	-	-	-	-	-	-	-	-	-	-	-	-	-	-	1	0.5876	1
	LIMK1	*ABCA1*	-	-	-	-	-	-	-	-	-	-	-	-	1	0.4929	1	1	1	2
	AGTR1	*NOS3*	-	-	-	-	-	-	1	0.1635	1	-	-	-	-	-	-	-	-	-
WS																				
	ARHGEF11	*ABCA1*	-	-	-	-	-	-	-	-	-	**1**	**0.0156**	**1**	1	0.2182	1	1	0.8156	7
	DLG5	*ABCA1*	-	-	-	-	-	-	-	-	-	**1**	**0.0156**	**1**	1	0.2182	1	1	0.8156	7
	LIN7A	*ABCA1*	-	-	-	-	-	-	1	0.6581	5	**1**	**0.0156**	**1**	1	0.2182	1	1	0.8156	7
	NFATC2	*ABCA1*	1	0.3247	2	1	0.4485	3	1	0.6581	5	**1**	**0.0156**	**1**	1	0.2182	1	1	0.8156	7
	IRS1	*GRB2*	2	0.3013	1	3	0.2321	1	6	0.3108	2	3	0.7552	2	5	0.2789	2	12	0.4088	3
	RPTOR	*IRS1*	1	0.3013	1	1	0.2321	1	1	0.3108	2	1	0.7552	2	1	0.2789	2	1	0.4088	3
	SMAD3	*IRS1*	1	0.3013	1	1	0.2321	1	1	0.3108	2	1	0.7552	2	1	0.2789	2	1	0.4088	3
	GRB2	*IRS1*	-	-	-	-	-	-	1	0.3108	2	1	0.7552	2	1	0.2789	2	1	0.4088	3
	GRIN2B	*IRS1*	-	-	-	1	0.2321	1	1	0.3108	2	-	-	-	1	0.2789	2	1	0.4088	3
	NOS3	*HSP90AA1*	-	-	-	1	0.2959	2	3	0.0537	1	-	-	-	2	0.6936	5	3	1	8
	EDNRB	*NOS3*	-	-	-	1	0.2959	2	1	0.0537	1	-	-	-	1	0.6936	5	1	1	8
	HSP90AA1	*NOS3*	-	-	-	-	-	-	1	0.0537	1	-	-	-	1	0.6936	5	1	1	8
	AGTR1	*NOS3*	-	-	-	-	-	-	1	0.0537	1	-	-	-	-	-	-	-	-	-
	TUB	*IRS1*	-	-	-	-	-	-	1	0.3108	2	-	-	-	1	0.2789	2	1	0.4088	3
	HNRNPU	*IRS1*	-	-	-	-	-	-	1	0.3108	2	-	-	-	-	-	-	1	0.4088	3
	TIMP3	*MMP3*	-	-	-	-	-	-	1	0.5179	3	-	-	-	-	-	-	1	0.7407	6
	CCR3	*CCL2*	-	-	-	-	-	-	1	0.5535	4	-	-	-	1	0.6722	4	1	0.2006	1
	LIMK1	*ABCA1*	-	-	-	-	-	-	-	-	-	-	-	-	1	0.2182	1	1	0.8156	7
	WNT3	*KL*	-	-	-	-	-	-	-	-	-	-	-	-	1	0.4262	3	1	1	9
	CCR1	*CCL2*	-	-	-	-	-	-	-	-	-	-	-	-	1	0.6722	4	1	0.2006	1
	DARC	*CCL2*	-	-	-	-	-	-	-	-	-	-	-	-	-	-	-	1	0.2006	1
	CCR2	*CCL2*	-	-	-	-	-	-	-	-	-	-	-	-	-	-	-	1	0.2006	1
	CCR5	*CCL2*	-	-	-	-	-	-	-	-	-	-	-	-	-	-	-	1	0.2006	1
	FN1	*LPA*	-	-	-	-	-	-	-	-	-	-	-	-	-	-	-	1	0.3628	2
	CANX	*LPA*	-	-	-	-	-	-	-	-	-	-	-	-	-	-	-	1	0.3628	2

Candidate gene	Interacting gene	Resident			Nearest			Adjacent			0.1Mbp			0.5Mbp			1Mbp		
		n	***p***	***r***	***n***	***p***	***r***	***n***	***p***	***r***	***n***	***p***	***r***	***n***	***p***	***r***	***n***	***p***	***r***
PHB	*IRS1*	-	-	-	-	-	-	-	-	-	-	-	-	-	-	-	1	0.4088	3
JAK2	*IRS1*	-	-	-	-	-	-	-	-	-	-	-	-	-	-	-	1	0.4088	3
TP53BP2	*IRS1*	-	-	-	-	-	-	-	-	-	-	-	-	-	-	-	1	0.4088	3
HSP90AB1	*IRS1*	-	-	-	-	-	-	-	-	-	-	-	-	-	-	-	1	0.4088	3
TYK2	*IRS1*	-	-	-	-	-	-	-	-	-	-	-	-	-	-	-	1	0.4088	3
RAD51	*IRS1*	-	-	-	-	-	-	-	-	-	-	-	-	-	-	-	1	0.4088	3
APOA1	*PON1*	-	-	-	-	-	-	-	-	-	-	-	-	-	-	-	2	0.4510	4
ITGB3	*CD36*	-	-	-	-	-	-	-	-	-	-	-	-	-	-	-	1	0.7304	5
COL1A1	*CD36*	-	-	-	-	-	-	-	-	-	-	-	-	-	-	-	1	0.7304	5
ITGB1	*CD36*	-	-	-	-	-	-	-	-	-	-	-	-	-	-	-	1	0.7304	5
BCAN	*MMP3*	-	-	-	-	-	-	-	-	-	-	-	-	-	-	-	1	0.7407	6
PLG	*MMP3*	-	-	-	-	-	-	-	-	-	-	-	-	-	-	-	1	0.7407	6
ACTN2	*NOS3*	-	-	-	-	-	-	-	-	-	-	-	-	-	-	-	1	1	8

Abbreviations: *n* – number of genes in search space that are interacting with gene. *p* – the *p*-value showing the statistical significance of the interaction as calculated by the Fishers-test. *r* – rank of gene based on *p*-value of interaction. *P*-values in bold are those that are significant.

Supp. Table S6. CAD PPI *ab initio* significant interactions and predictions for the MHS and MWS sets

Gene	Adjacent			0.5Mbp			1Mbp		
	n	***P***	***r***	***n***	***p***	***r***	***n***	***p***	***r***
MHS									
CLIP2	-	-	-	-	-	-	1	**0.0297**	1
CLASP1	-	-	-	-	-	-	1	0.0586	2
GTF2I	-	-	-	-	-	-	1	0.2786	3
USP7	-	-	-	-	-	-	1	1	4
MWS									
ELAVL2	1	**0.0305**	1	-	-	-	-	-	-
KLHDC10	1	0.7254	2	2	0.3328	19	5	0.3652	28
UBE2G2	-	-	-	9	**0.0137**	1	13	**0.0286**	2
GINS3	-	-	-	2	**0.0204**	2	2	0.0615	6
RNF157	-	-	-	1	**0.0342**	3	1	0.0621	7
MAP1LC3B	-	-	-	-	-	-	2	**0.0263**	1
ENTPD6	-	-	-	-	-	-	2	**0.0452**	3

Abbreviations: *n* – number of genes in search space that are interacting with gene. *p* – the *p*-value showing the statistical significance of the interaction as calculated by the Fishers-test. *r* – rank of gene based on *p*-value of interaction. *P*-values in bold are those that are significant.

Supp. Table S7. CAD PPI *ab initio* interactions for the WS sets

Gene	Resident			Nearest			Adjacent			0.1Mbp			0.5Mbp			1Mbp		
	n	*p*	*r*	*n*	*p*	*r*	*n*	*p*	*r*	*n*	*p*	*r*	*n*	*p*	*r*	*n*	*p*	*r*
JPH1	1	**0.0394**	1	1	0.0591	3	1	0.1423	25	1	0.0959	6	1	0.2719	76	1	0.4510	197
PLD1	2	**0.0476**	2	2	0.0979	5	3	0.1436	26	2	0.2161	14	3	0.7473	171	4	0.8106	310
NRP1	-	-	-	2	**0.0267**	1	3	**0.0243**	3	2	0.0656	3	5	**0.0073**	4	6	**0.0227**	13
SEMA3A	-	-	-	1	**0.0447**	2	1	0.1087	22	1	0.0728	5	1	0.2118	58	1	0.3622	167
SEMA3C	-	-	-	1	**0.0447**	2	1	0.1087	22	1	0.0728	5	1	0.2118	58	1	0.3622	167
PRDM4	-	-	-	-	-	-	2	**0.0041**	1	-	-	-	2	**0.0165**	7	2	0.0527	31
CDH2	-	-	-	-	-	-	5	**0.0148**	2	-	-	-	-	-	-	-	-	-
RPSA	-	-	-	1	0.3804	15	2	**0.0259**	4	3	0.4959	28	11	0.2354	65	24	0.3632	169
ERAP1	-	-	-	-	-	-	2	**0.0262**	5	-	-	-	2	0.0944	31	2	0.2533	122
EIF1	-	-	-	-	-	-	2	**0.0341**	6	-	-	-	2	0.1197	36	2	0.3085	144
SUPT3H	-	-	-	-	-	-	4	**0.0413**	7	-	-	-	6	**0.0468**	18	7	0.3230	149
MS4A2	-	-	-	-	-	-	2	**0.0427**	8	-	-	-	2	0.1464	41	2	0.3628	168
LAT2	-	-	-	-	-	-	-	-	-	2	**0.0198**	1	2	0.1464	41	2	0.3628	168
MACROD1	-	-	-	-	-	-	1	0.0739	15	1	**0.0491**	2	1	0.1467	42	1	0.2590	127
TRIM14	-	-	-	-	-	-	-	-	-	1	**0.0491**	2	1	0.1467	42	1	0.2590	127
GINS3	-	-	-	-	-	-	1	0.3929	66	-	-	-	5	**0.0020**	1	5	**0.0254**	14
VEGFA	-	-	-	-	-	-	-	-	-	-	-	-	6	**0.0050**	2	7	**0.0254**	15
SACM1L	-	-	-	-	-	-	-	-	-	-	-	-	1	**0.0070**	3	8	0.0837	41
DDX56	-	-	-	-	-	-	2	0.1798	31	-	-	-	3	**0.0094**	5	13	0.1383	67
UCHL1	-	-	-	-	-	-	-	-	-	-	-	-	6	**0.0119**	6	8	**0.0212**	12
MLH1	-	-	-	-	-	-	-	-	-	-	-	-	11	**0.0167**	8	15	0.0513	30
KPNA1	-	-	-	-	-	-	-	-	-	-	-	-	7	**0.0174**	9	9	0.0848	42
IPO5	-	-	-	-	-	-	-	-	-	-	-	-	5	**0.0182**	10	10	**0.0015**	3
ARNT2	-	-	-	-	-	-	-	-	-	-	-	-	3	**0.0185**	11	3	0.0878	43
BAIAP2	-	-	-	-	-	-	-	-	-	-	-	-	6	**0.0202**	12	6	0.2817	135
GPC1	-	-	-	-	-	-	-	-	-	-	-	-	3	**0.0262**	13	4	**0.0263**	16
LATS1	-	-	-	-	-	-	-	-	-	-	-	-	3	**0.0262**	13	3	0.1185	56
RERE	-	-	-	-	-	-	-	-	-	-	-	-	5	**0.0271**	14	7	**0.0322**	19
SIM2	-	-	-	1	0.0591	3	1	0.1423	25	-	-	-	2	**0.0314**	15	2	0.0958	51
ARHGAP44	-	-	-	-	-	-	1	0.1423	25	-	-	-	2	**0.0314**	15	2	0.0958	51
TRIM22	-	-	-	-	-	-	-	-	-	-	-	-	2	**0.0314**	15	2	0.0958	51
WDR47	-	-	-	-	-	-	-	-	-	-	-	-	2	**0.0314**	15	2	0.0958	51
POLR3A	-	-	-	1	0.7268	22	2	0.1799	32	1	0.2693	19	4	**0.0351**	16	13	0.1391	69
KALRN	-	-	-	1	0.2283	9	2	0.1327	24	-	-	-	4	**0.0359**	17	5	0.0761	39
ARC	-	-	-	-	-	-	1	0.1746	30	-	-	-	2	**0.0497**	19	2	0.1452	75

Gene	Resident			Nearest			Adjacent			0.1Mbp			0.5Mbp			1Mbp		
	n	***p***	***r***	***n***	***p***	***r***	***n***	***p***	***r***	***n***	***p***	***r***	***n***	***p***	***r***	***n***	***p***	***r***
VEGFB	-	-	-	-	-	-	-	-	-	-	-	-	2	**0.0497**	19	4	**0.0017**	4
CTR9	-	-	-	-	-	-	-	-	-	-	-	-	-	-	-	1	**0.0002**	1
ABCF2	-	-	-	-	-	-	-	-	-	-	-	-	-	-	-	2	**0.0007**	2
NSF	-	-	-	-	-	-	-	-	-	-	-	-	20	0.1849	53	32	**0.0076**	5
WASF1	-	-	-	-	-	-	2	0.3405	57	-	-	-	5	0.0926	29	10	**0.0093**	6
ERO1LB	-	-	-	-	-	-	-	-	-	2	1.0000	42	2	0.0907	28	4	**0.0150**	7
PLG	-	-	-	-	-	-	-	-	-	-	-	-	-	-	-	14	**0.0150**	8
PLAUR	-	-	-	-	-	-	-	-	-	-	-	-	-	-	-	8	**0.0167**	9
XPO5	-	-	-	-	-	-	-	-	-	-	-	-	-	-	-	2	**0.0196**	10
NCBP1	-	-	-	-	-	-	-	-	-	-	-	-	-	-	-	10	**0.0207**	11
CD82	-	-	-	-	-	-	-	-	-	-	-	-	-	-	-	8	**0.0212**	12
SORT1	-	-	-	-	-	-	-	-	-	-	-	-	1	1.0000	199	5	**0.0254**	14
CDK2	-	-	-	-	-	-	-	-	-	-	-	-	-	-	-	32	**0.0272**	17
SPATA5	-	-	-	-	-	-	-	-	-	-	-	-	1	0.1258	37	2	**0.0289**	18
ASAH1	-	-	-	-	-	-	-	-	-	-	-	-	-	-	-	1	**0.0363**	20
FYCO1	-	-	-	-	-	-	-	-	-	-	-	-	19	0.1767	51	34	**0.0380**	21
CASC5	-	-	-	-	-	-	-	-	-	-	-	-	1	0.3788	98	3	**0.0388**	22
PRKCH	-	-	-	-	-	-	1	0.5179	76	-	-	-	1	1.0000	199	6	**0.0390**	23
TGFBI	-	-	-	1	0.1414	6	2	0.0521	9	1	0.2228	15	2	0.1741	49	4	**0.0391**	24
ING1	-	-	-	-	-	-	-	-	-	-	-	-	-	-	-	2	**0.0401**	25
RABGAP1	-	-	-	-	-	-	-	-	-	-	-	-	-	-	-	27	**0.0432**	26
RANBP1	-	-	-	-	-	-	-	-	-	-	-	-	-	-	-	8	**0.0439**	27
ICT1	-	-	-	-	-	-	-	-	-	-	-	-	-	-	-	8	**0.0439**	27
CDC7	-	-	-	-	-	-	1	0.7240	94	-	-	-	8	0.0682	23	13	**0.0485**	28
TRMT112	-	-	-	-	-	-	-	-	-	-	-	-	-	-	-	6	**0.0493**	29

Abbreviations: *n* – number of genes in search space that are interacting with gene. *p* – the *p*-value showing the statistical significance of the interaction as calculated by the Fishers-test. *r* – rank of gene based on *p*-value of interaction. *P*-values in bold are those that are significant.

Supp. Table S8. CRT and MIR results for the CAD phenotype

Regulatory element	Nearest			Adjacent			0.5Mbp			1Mbp			Candidate gene(s)
	n	*p*	*r*	*N*	*p*	*r*	*n*	*p*	*r*	*n*	*p*	*r*	
MWS													
***Seeded* mode**													
**hsa-mir-126**	**1**	**0.0317**	**1**	1	0.1220	1	-	-	-	1	0.2051	1	*IRS1*
hsa-mir-145	1	0.0370	2	1	0.1412	2	-	-	-	1	0.2355	2	*IRS1*
OREG0005309	1	0. 0061	1	1	0. 0198	1	-	-	-	1	0. 0357	1	*IRS1*
WS													
***Seeded* mode**													
OREG0005309	1	0. 0190	1	1	0. 0562	1	-	-	-	1	0. 1467	1	*IRS1*
Ab initio mode													
**hsa-mir-181b-1**	-	-	-	**2**	**0.0552**	**1**	**3**	**0.0201**	**1**	3	0.0883	2	*CDX2, GATA6, AIDA*
**hsa-mir-181b-2**	-	-	-	**2**	**0.0552**	**1**	**3**	**0.0201**	**1**	3	0.0883	2	*CDX2, GATA6, AIDA*
hsa-mir-148a	-	-	-	-	-	-	2	0.0244	2	2	0.0704	1	*TGIF2-C20orf24, DNMT1*
hsa-mir-181c	-	-	-	-	-	-	2	0.0244	2	2	0.0704	1	*CDX2, GATA6*
hsa-mir-181a-1	-	-	-	-	-	-	2	0.1351	3	2	0.3226	5	*CDX2, GATA6*
hsa-mir-181a-2	-	-	-	-	-	-	2	0.1351	3	2	0.3226	5	*CDX2, GATA6*

Abbreviations: *n* – number of genes in search space that are regulated by a regulatory element. *p* – the *p*-value showing the statistical significance of the regulatory element as calculated by the Fishers-test. *r* – rank of regulatory element based on *p*-value. *P*-values in bold are those that are significant.

Supp. Table S9. CARDIoGRAMplusC4D study loci and candidate genes

Chromosome	SNP	Nearest gene(s)
1	rs4845625	*IL6R*
2	rs6544713	*ABCG5*
2	rs6544713	*ABCG8*
2	rs515135	*APOB*
2	rs2252641	*ZEB2*
2	rs2252641	*ACVR2A*
2	rs1561198	*GGCX*
2	rs1561198	*VAMP8*
4	rs7692387	*GUCY1A3*
4	rs1878406	*EDNRA*
5	rs273909	*SLC22A4*
5	rs273909	*SLC22A5*
6	rs10947789	*KCNK5*
6	rs4252120	*PLG*
7	rs2023938	*HDAC9*
8	rs264	*LPL*
8	rs2954029	*TRIB1*
13	rs9319428	*FLT1*
15	rs17514846	*FURIN*
15	rs17514846	*FES*
